# Ovarian steroid hormones: A long overlooked but critical contributor to brain aging and Alzheimer’s disease

**DOI:** 10.3389/fnagi.2022.948219

**Published:** 2022-07-19

**Authors:** Steven Jett, Eva Schelbaum, Grace Jang, Camila Boneu Yepez, Jonathan P. Dyke, Silky Pahlajani, Roberta Diaz Brinton, Lisa Mosconi

**Affiliations:** ^1^Department of Neurology, Weill Cornell Medical College, New York, NY, United States; ^2^Department of Radiology, Weill Cornell Medical College, New York, NY, United States; ^3^Department of Pharmacology, University of Arizona, Tucson, AZ, United States; ^4^Department of Neurology, University of Arizona, Tucson, AZ, United States

**Keywords:** hormones, menopause, estrogen, neuroimaging, Alzheimer’s disease, hormone therapy, menstrual cycle, pregnancy

## Abstract

Ovarian hormones, particularly 17β-estradiol, are involved in numerous neurophysiological and neurochemical processes, including those subserving cognitive function. Estradiol plays a key role in the neurobiology of aging, in part due to extensive interconnectivity of the neural and endocrine system. This aspect of aging is fundamental for women’s brains as all women experience a drop in circulating estradiol levels in midlife, after menopause. Given the importance of estradiol for brain function, it is not surprising that up to 80% of peri-menopausal and post-menopausal women report neurological symptoms including changes in thermoregulation (vasomotor symptoms), mood, sleep, and cognitive performance. Preclinical evidence for neuroprotective effects of 17β-estradiol also indicate associations between menopause, cognitive aging, and Alzheimer’s disease (AD), the most common cause of dementia affecting nearly twice more women than men. Brain imaging studies demonstrated that middle-aged women exhibit increased indicators of AD endophenotype as compared to men of the same age, with onset in perimenopause. Herein, we take a translational approach to illustrate the contribution of ovarian hormones in maintaining cognition in women, with evidence implicating menopause-related declines in 17β-estradiol in cognitive aging and AD risk. We will review research focused on the role of endogenous and exogenous estrogen exposure as a key underlying mechanism to neuropathological aging in women, with a focus on whether brain structure, function and neurochemistry respond to hormone treatment. While still in development, this research area offers a new sex-based perspective on brain aging and risk of AD, while also highlighting an urgent need for better integration between neurology, psychiatry, and women’s health practices.

## Introduction

### Sex is a genetic modifier of brain aging and risk of neurodegenerative disease

Sex differences in disease prevalence, manifestation, and response to treatment are rooted in genetic and hormonal differences between men and women. The effects of sex on neural aging phenotypes are often as large as, if not larger than the effects of other important variables (Cahill, 2006). In fact, female sex is the second greatest risk factor for late-onset Alzheimer’s disease (AD), second only to the aging process itself (Farrer et al., 1997). Moreover, susceptibility to aging-related neurodegenerative diseases and mental health conditions is greater in women than men, whereas men exhibit higher rates neuropsychiatric and learning disorders with developmental origins (Jazin and Cahill, 2010; McCarthy, 2016; Mauvais-Jarvis et al., 2020).

For decades, the general mindset was that sex differences in brain structure and function were controlled by a unitary program: genetic sex as the determinant of gonadal sex, and gonadal hormones as the determinants of brain sexual differentiation and subsequent neurophysiological and behavioral outcomes (McCarthy, 2016). Evidence has accumulated that numerous sex-specific factors, including hormonal, but also genetic and environment-driven epigenetic mechanisms, act in concert to provoke or eliminate sex differences in brain (McCarthy et al., 2009; Giatti et al., 2019). The combination of all these genetic and hormonal variables generates two different neurobiological systems in men and women. Starting at puberty, cells with androgen or estrogen receptors will be affected differently in men and women (McEwen, 2002), eliciting differences in disease predisposition, manifestation, and response to treatment. Overall, genetic sex is an important modifier of neurophysiology and neuropathology via genetic, epigenetic, and hormonal regulations (Cahill, 2006).

The value of understanding sex differences in brain aging and neurodegenerative disease is as self-evident as it is underappreciated. Historically, for multiple reasons, including the purported safety of women and their offspring, women of childbearing age were excluded from clinical trials (Clayton, 2016). As a result, for several decades, evidence-based medicine was defined by male physiology. In 1993, the US National Institutes of Health (NIH) mandated the inclusion of women in NIH-funded clinical trials, but many investigators did not follow this mandate (Schiebinger et al., 2016). This was followed by a 2014 mandate to consider sex as a biological variable in basic research (Mazure and Jones, 2015). However, based on arguments that ovarian hormone fluctuations made female animals too volatile to assess, preclinical research and drug development studies have also predominantly used male animal models (McCarthy et al., 2017). Today, even though women are included in biomedical research, the data from both clinical trials and research studies is rarely broken down by sex.

The field of cognitive aging and AD is no exception as sex and gender are more likely to be used as confounders than predictors. As of 2022, of all clinical trials of AD, none has set out to examine sex differences in efficacy or outcomes (Ferretti et al., 2018). Recent research, however, has elucidated the important neuroprotective role of ovarian steroid hormones and their receptors for cognitive aging and AD (Morrison et al., 2006; Brinton et al., 2015).

### Focus of this review

This review explores the role of ovarian steroid hormones, especially 17β-estradiol, as contributors of brain aging and risk of AD or dementia. Endogenous exposures to ovarian hormones include chiefly pubertal timing, the menstrual cycle, pregnancy, and menopause. Exogenous hormonal exposures include chiefly use of hormonal therapy such as oral contraceptives and menopause hormone therapy (MHT). Throughout the review, the emphasis is on studies that used brain biomarkers of AD, primarily brain imaging, conducted in cisgender women with sound methodology.

### Search strategy and selection criteria

We conducted a systematic review of neuroimaging studies of the menstrual cycle, oral contraceptives, menopausal status, and randomized clinical trials of MHT, as well as of imaging studies of pubertal timing and pregnancy as related to cognitive aging and AD risk. We also provide a narrative review of psychometric studies of all exposures. We searched PubMed and the Web of Science for papers published in English between 1998 and 2022, using “estrogen,” “sex steroids,” “ovarian hormones,” or “sex hormones,” all exposures and outcomes as search terms. Although we tried to cite seminal studies as necessary, because of space limitation, representative reviews were also selected. We also provide general information on the actions of ovarian steroid hormones in brain to provide context for the research findings linking these hormones to brain aging and AD risk.

## Action of ovarian steroid hormones in brain

### The brain is a target for ovarian steroid hormones

The primary hormones secreted by the ovaries are 17β-estradiol (estradiol, E2), the most prevalent form of estrogen produced before menopause, and progesterone, a type of progestagen. Both hormones pass through the blood–brain barrier and have receptors throughout the brain (McEwen, 2002). As reviewed below, estradiol receptors (chiefly ERα and ERβ) are present throughout areas of the brain involved in both reproductive and cognitive functions (McEwen et al., 2001). However, there is controversy regarding estrogen receptor expression across species, especially ERβ, due to limited ERβ antibody specificity (Maioli et al., 2021). Validated techniques have confirmed ERβ in rodent but not human brain (Maioli et al., 2021). Development of ERβ antibodies with higher binding specificity is needed to resolve this inconsistency, as discussed elsewhere (Andersson et al., 2017). Moreover, despite animal research demonstrating the presence of progesterone receptors (PRA and PRB) in brain regions involved in cognition, little is known about their location or function in the human brain (Brinton et al., 2008). As such, here we focus primarily on the action of estradiol on brain structure and function.

Estradiol is a steroid hormone synthesized in a series of enzymatic steps beginning with the conversion of cholesterol into pregnenolone in the mitochondria (McEwen, 2002). The final enzymatic step, the conversion of testosterone into estradiol, is catalyzed by the enzyme aromatase, or estrogen synthase (McEwen, 2002). In neurons and astrocytes, depending on tissue and time period, estradiol can also be synthesized from androstenedione and estrone (E1) (Cui et al., 2013). Starting at puberty, and for the duration of a woman’s reproductive life, estradiol is mainly produced in the ovary. Its levels in plasma change during development, fluctuate cyclically during the menstrual cycle, increase dramatically during pregnancy, drop during lactation, and eventually decline after menopause (McEwen, 2002).

Estradiol is also locally synthesized in different tissues, including brain. Recent research demonstrates that the brain is a steroidogenic organ (Arevalo et al., 2015) expressing the molecules and enzymes necessary for the conversion of endogenous cholesterol into local estradiol. As a result, the brain is a target for the action of both peripheral estradiol and neuroestradiol, e.g., estradiol synthesized in neural cells (Arevalo et al., 2015). There is emerging evidence that both types of estradiol, from ovaries and brain, control various neurobiological processes, including sexual behavior, but also neurological functions such as regulation of body temperature and blood pressure, response to stress, some aspects of mood and of cognition (Lupien et al., 2009). Importantly, brain steroidogenesis is regulated independently of peripheral steroidogenesis, and brain steroid levels do not correlate with plasma steroid levels in animals (Caruso et al., 2013).

Moreover, there is some evidence that the brain upregulates synthesis of neurosteroids in response to the drop in estrogen following oophorectomy as a compensatory adaptive reaction (Caruso et al., 2010). This suggests that similar mechanisms might be in place in response to naturally occurring declines in ovarian hormones following spontaneous menopause, though this remains to be confirmed.

### Estradiol: The “master regulator” of the female brain

Estradiol has been called the “master regulator of the female brain” (Rettberg et al., 2014) due to its wide range effects on neuronal structure and function. Its neuroprotective role is of particular relevance for cognitive aging and AD. In mouse models of AD, decreasing estradiol levels in plasma following oophorectomy exacerbate brain damage under neurodegenerative conditions (Azcoitia et al., 1999), trigger decrease cerebral glucose metabolism (CRMglc) (Ding et al., 2013), and increase amyloid-β fibrillization (Yue et al., 2005).

Estrogen therapy reduces such damage (Azcoitia et al., 1999), normalizing CMRglc and reducing Aβ oligomers in oophorectomized mice (Yue et al., 2005). Estradiol’s neuroprotective action may be related to its role in maintaining metabolic homeostasis in body and brain (Frank et al., 2014; Rettberg et al., 2014). In brain, estradiol regulates glucose metabolism, glycolysis, oxidative phosphorylation and subsequent ATP generation in neurons (Rettberg et al., 2014). Substantial evidence indicates that metabolic alterations play a role in neurodegenerative diseases including AD (Lin and Beal, 2006).

Additionally, genetic studies have identified variants of the gene encoding for the aromatase enzyme that are associated with an increased risk for AD (Iivonen et al., 2004; Huang and Poduslo, 2006; Medway et al., 2014) These genetic variants may result in decreased estradiol synthesis in brain, which, together with decreased serum estradiol levels in post-menopausal women may increase the risk for AD (Huang and Poduslo, 2006). Aromatase expression is indeed increased in prefrontal cortex of patients with severe AD, a phenomenon that has been interpreted to be part of a “rescue program” (Luchetti et al., 2011).

Estrogen receptors (ERs) also coordinate multiple neuroprotective signaling cascades, either via direct activation or by the interaction of ERs with the receptors for other neuroprotective factors. Estradiol action in brain can be both delayed in onset and prolonged in duration (“genomic”) or rapid in onset and short in duration (“non-genomic”) (McEwen and Milner, 2017). Both ERα and ERβ are expressed in regions including hippocampus, amygdala, and hypothalamus, their distribution density differs. ERα shows greater distribution in hypothalamic nuclei associated with sexual behavior, whereas ERβ is expressed more in areas associated with cognition such as basal forebrain, prefrontal cortex, temporal and parietal regions, and posterior cingulate (Foster, 2012). Additionally, while both ERα and ERβ participate in the overall neuroprotective action of the estradiol, ERα is more closely involved in neuroprotection, as demonstrated by animal models of focal ischemia (Dubal et al., 2001), whereas ERβ has been shown to be involved in cognition, thought to promote learning and memory, neural plasticity, and regulating neurotrophic factors (Zhao et al., 2015). The G-protein coupled estrogen receptor (GPER1) shows widespread brain distribution, with heavy concentration in key brain regions including hippocampus and amygdala (Hadjimarkou and Vasudevan, 2018) and play a key role in mediating the rapid action of estradiol.

ERα and ERβ are also implicated in modulating the immune system. Both receptors are expressed on microglia and astrocytes, both involved in neuroinflammation and implicated in Alzheimer’s disease (Mishra and Brinton, 2018). Activation of ERα and ERβ via estradiol treatment has been reported to decrease inflammatory responses such as phagocytosis and cytokine secretion, ultimately having an anti-inflammatory and neuroprotective effect (Mishra and Brinton, 2018). Activation of ERα has also been reported to shorten the inflammatory response to infection in preclinical studies (Villa et al., 2015). There is increasing evidence that chronic inflammatory processes are activated during midlife chronological and endocrine aging, which ultimately limit the clearance capacity of microglia and lead to immune senescence (Mishra and Brinton, 2018). The inflammatory immune response is a possible unifying factor that bridges across the three major risk factors for AD in women: aging, menopause, and ApoE epsilon 4 (ApoE4) genotype (Mishra and Brinton, 2018).

### Influence of sex hormones across a woman’s life

Ovarian hormones affect the nervous system in ways that extend beyond their essential actions of regulating gonadotropin secretion and modulating sexual behavior. As reviewed below, at a neurological level, estrogens are involved in regulation of thermoregulation, mood, sleep, and cognitive abilities, among other factors (McEwen et al., 1997). From a cognitive aging perspective, both estradiol and progesterone influence verbal memory, fluency, performance on spatial tasks, and fine motor skills (Maki and Henderson, 2016). Declines in these hormones with menopause have been associated with an increased risk of cognitive impairment, affective disorders, and AD pathology (Rahman et al., 2019; Jett et al., 2022).

In what follows, we review research elucidating the role of ovarian steroid hormones in cognitive aging and AD risk across the female lifespan, including studies of puberty, menstrual cycle, hormonal contraceptive use, the menopause transition, and hormone therapy for menopausal symptoms.

## Pubertal timing and menstrual cycle

Puberty is characterized by surges in the production of sex hormones, which in turn prompt dramatic organizational changes in the brain, followed by transformative changes in cognition and behavior (Sisk and Foster, 2004). For girls, the maturation of the ovaries with the subsequent production of estradiol and progesterone typically occurs around age 11–12 years, ranging from 10 to 18 years (Anderson et al., 2003). This results in the development of secondary sexual characteristics and of menarche, or the first menstrual bleeding.

There is ample evidence that ovarian sex hormones influence brain development and cognition during adolescence. While reviewing these findings is beyond the scope of this review, we recommend prior reviews on the topic (Giedd et al., 1999; Sisk and Foster, 2004; Blakemore, 2008). Herein, we focus on links between early hormonal exposures and cognitive aging in midlife and older age. Of all the factors involved in puberty and adolescence, two have been consistently examined as possible predictors of future cognitive impairment and AD or dementia: pubertal timing and the menstrual cycle.

### Pubertal timing and age at menarche

A recent hypothesis posits that the brain has declining sensitivity to sex hormones throughout adolescence, such that females who mature early have greater effective ovarian hormone exposure than those who mature late (Schulz et al., 2009). The age at which a woman enters menarche has gained attention for a possible relationship with cognition in later life due to longer estrogen exposure when menarche occurs at a younger age (Bernstein et al., 1991). While research on this topic is scant, some studies indicate associations between an early age at menarche and greater white matter integrity in frontal cortex in adolescence (Chahal et al., 2018). Thus, pubertal timing may facilitate brain maturation due to longer exposure to ovarian sex hormones, which may in turn confer greater brain reserve later in life.

Nonetheless, the majority of studies so far indicate null associations between age at menarche and cognitive impairment or AD risk (Geerlings et al., 2001; Henderson et al., 2003; Colucci et al., 2006; Fox et al., 2013; Prince et al., 2018; Najar et al., 2020; Song X. et al., 2020). On the other hand, in some studies, a younger age at menarche correlated with better visual memory performance on Benton’s visual retention test and psychomotor speed on a trail making task (task A) (Ryan et al., 2009), and with a reduced risk of dementia or AD in later life (Rasgon et al., 2005a; Gilsanz et al., 2019). Additionally, the Gothenburg H70 Birth Cohort study reported associations between a younger age at menarche and lower CSF Aβ_42/40_ ratio and higher hyperphosphorylated tau levels among older post-menopausal women free of dementia (Najar et al., 2021). More studies of pubertal timing, ideally spanning puberty and young adulthood to midlife and beyond, and including the use of AD biomarkers, are needed to clarify the strength and reproducibility of these associations.

### Menstrual cycle

The typical menstrual cycle is 28 days long, with normal variation ranging from 22 to 35 days (Reed and Carr, 2000; Grieger and Norman, 2020). Menstruation is generally considered the beginning of the cycle, which is divided into two phases, follicular and luteal. The follicular phase begins after the first day of menstruation and is characterized by initial low levels of both estradiol and progesterone followed by rising estradiol. Estradiol levels peak before ovulation (∼day 14), triggering the release of luteinizing hormone (LH). The luteal phase begins after ovulation and is characterized by a decrease in estradiol that settles at moderate levels, while progesterone begins to rise. If the egg is not fertilized, estradiol and progesterone decline during the second half of the phase (i.e., premenstrual phase), triggering menstruation and a new cycle. As these phases are relatively easy to pinpoint, studies of the menstrual cycle offer a unique opportunity to clarify the influence of ovarian hormones on neuronal circuits implicated in the regulation of cognitive and emotional processing.

Seminal animal studies from the early 1990’s demonstrated that estradiol levels regulate synaptogenesis and synapse density on excitatory spines in hippocampal CA1 pyramidal neurons in female rats (Woolley and McEwen, 1992), which have been since replicated by many investigators (for example, Hara et al., 2015; McEwen and Milner, 2017; Sheppard et al., 2019). Fluctuations in synaptogenesis occur throughout the estrous cycle, with increases in synapses on dendritic spines after estrogen treatment, along with decreases in spine synapse density that occurs between the days of proestrus and estrus (Woolley and McEwen, 1992). Consistent with these observations, neuroimaging and cognitive studies provide evidence for changes in brain structure, function, and cognitive performance across the menstrual cycle or as a function of ovarian hormones.

The long-held view is that verbal memory and implicit memory are enhanced in the late follicular and midluteal phase, when estradiol is high (Hampson, 1990; Maki et al., 2002; Pletzer et al., 2011), whereas spatial and numerical abilities are enhanced in the early follicular phase, when estradiol is low (Hausmann et al., 2000; Courvoisier et al., 2013). Nonetheless, results are generally inconsistent (Sacher et al., 2013; Sundström Poromaa and Gingnell, 2014). Specifically for brain aging and AD, only one study to date has investigated possible associations of menstrual cycles and AD risk (Fox et al., 2013). In a cohort of 89 elderly British women, Fox et al. (2013) reported a marginally significant association between the number of menstrual cycles, defined as the number of months between menarche and menopause, free from oral contraceptive use, pregnancy, breastfeeding, and post-partum anovulation, and a lower risk of AD. Each additional month of having a menstrual cycle corresponded to a 0.3% reduction in risk of AD.

**FIGURE 1:**
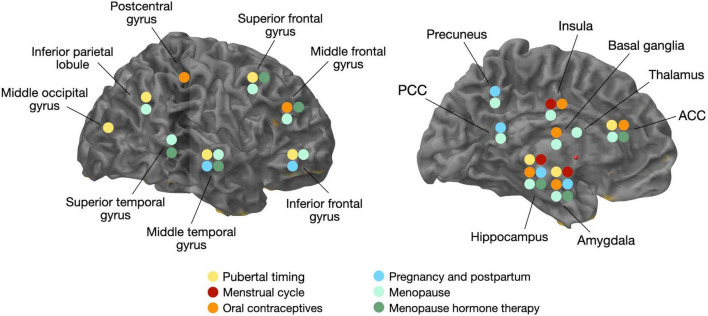
Overview of volumetric gray matter changes related to changes in estradiol levels and/or hormonal transitions. ACC, anterior cingulate cortex; PCC, posterior cingulate cortex.

Neuroimaging studies of the menstrual cycle are summarized in Table 1. Several structural MRI studies report changes in the volume of several cortical and subcortical regions across the menstrual cycle (Figure 1). Most studies indicate increased hippocampal or amygdala volumes during the late follicular phase, when estradiol levels are rising and progesterone is low (Protopopescu et al., 2008; De Bondt et al., 2013a; Lisofsky et al., 2015; Pletzer et al., 2018), with some exceptions (Ossewaarde et al., 2013). Two studies also demonstrate a direct association between higher estradiol levels and larger hippocampal volume (Barth et al., 2016; Pletzer et al., 2018), while another study found a positive association between estradiol levels and the volume of another limbic structure, the parahippocampal gyrus (Lisofsky et al., 2015). Insular volume has also been reported, being positively associated with estradiol levels and higher during the follicular phase (De Bondt et al., 2016). Prefrontal cortex volume and thickness also appear to be positively associated with estradiol levels (Dubol et al., 2021).

**TABLE 1:** Summary of studies investigating the effects of the menstrual cycle and of use of hormonal contraceptives on neuroimaging outcomes.

Study	Participants	Age, years	Exposure	Neuro-imaging technique	Cognitive measures	Study design	Main findings
Marečková et al. (2012)	10 naturally cycling women, 10 OC users	18–29	Menstrual cycle, OC	fMRI	Emotional processing	Repeated measures analysis	OC users exhibited higher BOLD signals in right FFA to ambiguous and angry faces vs. naturally cycling women Higher BOLD signal in FFA when observing angry faces during menstruation Longer OC duration was associated with higher BOLD signal in left FFA during ambiguous and angry face conditions
De Bondt et al. (2013b)	15 naturally cycling women, 15 OC users	18–28	Menstrual cycle, OC use	DTI	–	Repeated measures analysis	Higher mean diffusivity in fornix in OC users vs. naturally cycling women during the luteal phase Mean diffusivity in fornix was negatively associated with luteinizing hormone and estradiol
Bayer et al. (2014)	22 naturally cycling women	19–33	Menstrual cycle	fMRI	Emotional processing	Repeated measures analysis	No effect of menstrual cycle on recognition accuracy Higher recollection performance for negative items during early follicular phase vs. luteal phase Greater activity in HIP and ACC during both positive and negative emotional stimuli during early follicular phase vs. luteal phase Greater activity in bilateral ACC during positive emotional stimuli during early follicular phase vs. luteal phase Greater activity in left AMY during negative emotional stimuli during luteal phase vs. early follicular phase
Gingnell et al. (2014)	16 naturally cycling women, 17 women with PMDD	34 (9)	Menstrual cycle	fMRI	Emotional processing	Repeated measures analysis	No cycle phase difference on functional connectivity PMDD women rated social stimuli as more negative than controls during luteal phase PMDD women exhibited higher activity in AMY and insula, and lower activity in ACC toward social stimuli than controls during luteal phase. No group differences during follicular phase
Hjelmervik et al. (2014)	16 naturally cycling women	23 (5)	Menstrual cycle	Resting state fMRI	–	Repeated measures analysis	No effects of menstrual cycle on resting state connectivity
Petersen et al. (2014)	46 women using OCs (22 during placebo pill phase; 24 during active pill phase) vs. 45 naturally cycling women (20 in early follicular phase 25 in midluteal phase)	18–40	Menstrual cycle and OC use	Resting state fMRI	–	Group comparisons of menstrual cycle phases and OC pill phases	Greater connectivity of DMN regions in early follicular vs. midluteal phase and vs. OC users Greater connectivity of ECN regions in early follicular vs. midluteal phase and active OC users Greater connectivity of ECN regions in placebo vs. active OC users
Pletzer et al. (2014)	14 women using combined OCs vs. 16 naturally cycling women	25 (5)	OC use	fMRI	Number tasks	Group comparisons	Lower FPN activation in OC users vs. natural cyclers in follicular phase Greater PFC and inferior parietal lobe activation in OC users vs. natural cyclers in midluteal phase
Thimm et al. (2014)	21 naturally cycling women	18–34	Menstrual cycle	fMRI and rsfMRI	Cognitive control/attention	Repeated measures analysis	Greater ACC activity during menstrual and late follicular vs. midluteal phase Greater connectivity between FPN regions during menstrual vs. luteal phase
Albert et al. (2015)	28 naturally cycling women	18–45	Menstrual cycle	fMRI	Montreal Imaging Stress Task	Between-group comparison	Greater left HIP activity during psychosocial stress during ovulation vs. early follicular phase Bilateral HIP activity during stress was positively associated with estradiol levels
Biegon et al. (2015)	10 PRE, 10 POST	23–67	Menstrual cycle, menopause status	^11^C-vorozole PET	–	Group comparison	Aromatase activity did not differ between cycle phases POST had lower Aromatase enzyme activity vs. PRE
De Bondt et al. (2015a)	10 naturally cycling women, 21 OC users	18–30	Menstrual cycle, OC use	^1^H MRS	–	Repeated measures analysis	Higher GABA+/Cr ratios in PFC during ovulation vs. follicular phase, luteal phase, active and inactive OC phase No difference in GABA+/Cr ratios in PFC between active or inactive OC phase vs. follicular or luteal phases No difference in GABA+/Cr ratios in PFC between active vs. inactive OC phase
De Bondt et al. (2015b)	19 women using monophasic OCs vs. 18 naturally cycling women	24 (3)	Menstrual cycle and OC use	Resting state fMRI	–	Repeated measures analysis and group comparisons	No effects of menstrual cycle or OC use on resting state connectivity
Diekhof and Ratnayake (2016)	15 naturally cycling women	25 (2)	Menstrual cycle	fMRI	Reinforcement learning	Repeated measures analysis	Greater ACC activity to negative feedback during midluteal vs. late follicular phase ACC activity correlated with avoidance learning during midluteal phase Greater avoidance learning during midluteal vs. late follicular phase
Franke et al. (2015)	7 naturally cycling women	21–31	Menstrual cycle	Structural MRI, BrainAGE	–	Repeated measures analysis	No differences in GMV, WMV or CSF volume over the menstrual cycle Lower BrainAGE scores during ovulation vs. menses Higher estradiol levels associated with lower BrainAGE scores
Frokjaer et al. (2015)	60 naturally cycling women	24 (5)	Menstrual cycle	[^11^C]DASB PET	–	Double-blind, randomized placebo-controlled study	No changes in serotonin activity Increases in depressive symptoms correlated positively with increase in serotonin binding within the GnRHa treated group
Henningsson et al. (2015)	56 naturally cycling women	24 (5)	Menstrual cycle	fMRI	Emotional processing	Double-blind, randomized placebo-controlled study	No effects of GnRHa vs. placebo
Jacobs et al. (2015)	13 naturally cycling women, 11 women with remitted MDD	43–50	Menstrual cycle	fMRI	Emotional processing	Repeated measures analysis	Reduced brain activity in left HIP, right AMY and hypothalamus during late follicular phase vs. early follicular phase in healthy controls after stress challenge No differences in brain activity for MDD women between early or late follicular phase
Lisofsky et al. (2015)	21 naturally cycling women (11 controls; 10 PMDD patients),	22–31	Menstrual cycle	Structural MRI	–	Repeated measures analysis	Larger HIP GMV in late vs. early follicular phase Estradiol levels positively correlated with PHG GMV
Petersen et al. (2015)	21 women in follicular phase, 25 women in luteal phase, 22 OC users in active phase, 22 OC users in inactive phase	18–40	Menstrual cycle, OC use	Structural MRI	–	Group comparison	Larger global GMV in naturally cycling women vs. OC users PCC and orbitofrontal cortex thickness greater in naturally cycling women vs. OC users Greater cortical thickness in follicular phase, luteal phase, and OC inactive phase vs. OC active phase
Pletzer et al. (2015)	22 women using antiandrogenic OCs vs. 18 women using androgenic OCs vs. 20 naturally cycling women in menstrual or early follicular phase	25 (6)	OC use	Structural MRI	–	Group comparisons	Larger FFA and PHG GMV in users of antiandrogenic OCs vs. naturally cycling women Smaller frontal GMV in users of androgenic OCs vs. naturally cycling women No group differences in HIP, PHG, and ACC
Zhu et al. (2015)	10 naturally cycling women	18–38	Menstrual cycle	fMRI	Mental rotation task	Repeated measures analysis	Greater left superior parietal cortex activity during late follicular phase associated with decreased errors in mental rotation task vs. early follicular phase Greater right superior parietal and superior frontal cortex activity associated with longer reaction time during late follicular phase vs. early follicular phase
De Bondt et al. (2016)	24 naturally cycling women, 23 androgenic OC users, 10 anti-androgenic OC users	18–34	Menstrual cycle, OC use	Structural MRI	–	Repeated measures analysis	Larger insula GMV during ovulation vs. luteal phase No differences between androgenic OC users vs. anti-androgenic OC users No difference between follicular phase vs. OC use Somatic premenstrual symptoms were associated with frontal cortex GMV in androgenic OC users
Lisofsky et al. (2016)	28 naturally cycling women, 28 OC users	16–33	Menstrual cycle, OC use	Structural MRI, rsfMRI	Emotional processing, episodic verbal memory, working memory, spatial memory	Repeated measures analysis	Lower positive affect in OC users vs. naturally cycling women No changes in cognitive performance in either group Lower left AMY and PHG volume in OC users vs. naturally cycling women Negative functional connectivity between AMY, PHG and DLPFC in OC users vs. naturally cycling women
Pletzer et al. (2016)	16 women using androgenic OCs vs. 16 using antiandrogenic OCs vs. 18 naturally cycling women	25 (6)	Menstrual cycle and OC	rsfMRI	–	Repeated measures and group comparisons	Greater temporal-to-DMN connectivity during late follicular vs. menstrual/early follicular phase Greater connectivity of DMN during midluteal phase vs. menstrual/early follicular phase Increased PFC-to-DMN connectivity in androgenic OC users vs. menstrual/early follicular phase Increased basal ganglia-to-DMN connectivity in antiandrogenic OC users vs. menstrual/early follicular phase
Arnoni-Bauer et al. (2017)	18 naturally cycling women, 11 OC users	18–35	Menstrual cycle, OC	fMRI	–	Repeated measures analysis	Greater activity in AMY, ACC, insula, and hypothalamus during luteal phase and OC users vs. follicular phase No difference for OC users
Syan et al. (2017)	25 naturally cycling women	16–45	Menstrual cycle	rsfMRI	–	Repeated measures analysis	No differences in connectivity between menstrual cycle phases Progesterone positively correlated with connectivity of FPN and DMN regions in late luteal phase
Donishi et al. (2018)	93 naturally cycling women	18–24	Menstrual cycle	rsfMRI	–	Group comparison	Higher percentage of global hubs in frontal medial cortex during the follicular phase vs. luteal phase Global hubs in sensorimotor cortex were greater during luteal phase vs. follicular phase
Engman et al. (2018)	18 naturally cycling women, 17 OC users, all who had previously experienced OC-related negative affect	25 (4)	Menstrual cycle, Oral contraceptives	fMRI, rsfMRI	–	Double-blind, randomized placebo-controlled trial	Naturally cycling women exhibited higher RSFC in AMY to middle and superior frontal gyri, paracentral lobule, and cerebellum, and higher RSFC in dorsal ACC to middle frontal, superior and transverse temporal, postcentral gyri, during the luteal phase vs. follicular phase OC users exhibited higher dorsal ACC RSFC in superior frontal gyrus and precuneus and lower RSFC in AMY to postcentral gyrus during treatment vs. follicular phase Naturally cycling placebo users exhibited higher AMY RSFC in postcentral gyrus and cuneus vs. OC users during treatment
Hjelmervik et al. (2018)	15 naturally cycling women	23 (5)	Menstrual cycle	^1^H MRS	–	Repeated measures analysis	Higher creatine levels in left PFC vs. right PFC during follicular and menstrual phases, no hemisphere differences during luteal phase
Petersen et al. (2018)	18 naturally cycling women, 18 women with PMDD	18–41	Menstrual cycle	fMRI	Emotion regulation task	Repeated measures analysis	Women with PMDD exhibited lower negative emotion regulation during the luteal phase vs. follicular phase or naturally cycling luteal phase Lower activation in right DLPFC during emotion regulation task in women with PMDD during luteal phase vs. follicular phase and naturally cycling women in luteal phase No group or cycle phase differences in AMY activation
Pletzer et al. (2018)	55 naturally cycling women	18–35	Menstrual cycle	Structural MRI	–	Repeated measures analysis	Larger HIP GMV in late follicular phase vs. menstrual/early follicular and midluteal phases, which positively correlated with estradiol levels Greater basal ganglia GMV in menstrual/early follicular vs. late follicular phase, which positively correlated with progesterone levels
Dan et al. (2019)	20 naturally cycling women	21–29	Menstrual cycle	fMRI	Emotional face matching task	Repeated measures analysis	No significant difference between brain activation to negative emotional faces between mid-follicular vs. late-luteal phases
Petersen et al. (2019)	18 naturally cycling women, 17 women with PMDD	18–44	Menstrual cycle	fMRI	Emotion-regulation task	Repeated measures analysis	No effect of menstrual phase on resting-state functional connectivity Greater connectivity between middle temporal cortex and left ECN in PMDD women vs. controls Greater connectivity between left AMY and PCC, mid-cingulate cortex, and right angular gyrus, and between right AMY and middle temporal cortex during follicular phase vs. luteal phase
Pletzer et al. (2019a)	131 naturally cycling women (79 past OC users, 52 non-users)	18–35	Previous OC use	Structural MRI	–	Group comparison	No GMV difference between OC past users and non-users Positive association between past OC duration and bilateral HIP and basal ganglia GMV Negative association between time since OC discontinuation and bilateral HIP and basal ganglia GMV Associations between OC duration and HIP GMV non-significant after controlling for time since OC discontinuation No difference between androgenic vs. anti-androgenic OC
Pletzer et al. (2019b)	36 naturally cycling women	25 (4)	Menstrual cycle	fMRI	Spatial navigation and verbal fluency	Repeated measures analysis	Increased HIP/PHG activity in preovulatory phase during navigation and fluency, which positively correlated with estradiol levels Increased caudate and DLPFC activity in midluteal phase during navigation and fluency, which positively correlated with progesterone levels
Sundström Poromaa et al. (2019)	90 naturally cycling women	18–49	Serum Allopregnanolone	^11^C DASB PET	–	Group comparison	Negative association between serum allopregnanolone levels and serotonin binding in PFC
Şafak (2019)	32 naturally cycling women	20–40	Menstrual cycle	ADC	–	Group comparison	No differences between follicular phase vs. luteal phase
Weis et al. (2019)	19 naturally cycling women	18–34	Menstrual cycle	rsfMRI	–	Repeated measures analysis	Greater frontal-to-DMN connectivity during menstrual/early follicular vs. late follicular phase
Herrera et al. (2020)	20 OC users	18–28	OC use	fMRI	n-back working memory task	Repeated measures analysis	Greater task load-related deactivation in frontal pole, PCC, and middle temporal gyrus during hormone-present phase vs. hormone-absent phase
Hidalgo-Lopez et al. (2020)	60 naturally cycling women	18–35	Menstrual cycle	rsfMRI	–	Repeated measures analysis	Decreased intrinsic connectivity in the right angular gyrus with medial prefrontal and posterior cingulate/precuneus areas during the luteal phase vs. pre-ovulatory phase Increased HIP EC during luteal phase vs. pre-ovulatory phase Higher ALFF in caudate during luteal phase vs. pre-ovulatory phase and menses Increased connectivity between right caudate and right middle frontal gyrus during pre-ovulatory phase vs. menses Increased connectivity between left putamen and contralateral dorsomedial thalamus during luteal phase vs. menses
Larsen et al. (2020)	16 OC users, 37 non-users (8 with IUD)	26 (5)	OC use	^11^C SB207145-PET	–	Cross-sectional	9–12% reduced global serotonin binding in OC users vs. non-users, with largest difference in HIP
Meeker et al. (2020)	14 naturally cycling women	18–45	Menstrual cycle	fMRI, rsfMRI, and structural MRI	–	Repeated measures analysis	Greater GMV in parietal cortex during menstrual phase vs. follicular, ovulatory, and luteal phases Greater parietal WMV during ovulatory and luteal phases vs. follicular and menstrual phases Greater primary somatosensory cortex GMV during menstrual phase vs. follicular phase Greater WMV in right hemisphere during follicular phase vs. luteal phase Greater functional connectivity between left IPL and right visual cortex during ovulatory phase vs. luteal phase Greater functional connectivity between right and left IPL during ovulatory phase vs. follicular phase Greater functional connectivity between right IPL and left medial PFC during luteal phase vs. menstrual phase
Nasseri et al. (2020)	24 OC users (monophasic HC)	18–35	OC use	rsfMRI	–	Repeated measures analysis	Greater functional connectivity between left AMY and right VMPFC during hormone-present phase vs. hormone-absent phase after a stress test Greater functional connectivity between left PHG and right superior lateral occipital cortex during hormone-absent phase vs. hormone-present phase No differences in HIP functional connectivity between hormone-present phase vs. hormone-absent phase
Sharma et al. (2020)	48 naturally cycling women, 27 OC users	18–26	OC use	fMRI and structural MRI, DTI	Emotional n-back test	Group comparison	Lower GMV in right putamen in OC users vs. naturally cycling women Higher WMV in left PHG, HIP, right AMY, putamen, and rectus in OC users vs. naturally cycling women Higher FA in left HIP in OC users vs. naturally cycling women Higher brain activity in left lingual gyrus, paracentral lobule, right insula, frontal cortex, supplementary motor area in OC users vs. naturally cycling women during negative stimuli memory task No group difference in errors made during memory task
Zhuang et al. (2020)	16 naturally cycling women	20–24	Menstrual cycle	fMRI and rsfMRI	Intertemporal binary choice task	Repeated measures analysis	Greater activation in bilateral lingual gyrus, calcarine gyrus, left middle and inferior occipital gyri during the mid-luteal phase vs. late follicular phase More activity in left putamen, HIP, insula, bilateral caudate and visual areas during delay discounting during late follicular phase vs. mid-luteal phase Greater activity in bilateral putamen when choosing short-term reward during late follicular phase vs. mid-luteal phase During the late follicular phase, greater dorsal striatum activity was associated with short-term reward choices. During the mid-luteal phase, greater DLPFC activity was associated with delayed reward choices Greater functional connectivity between left putamen and DLPFC during the mid-luteal phase vs. late follicular phase
Zhuang et al. (2020)	49 naturally cycling women	19–28	Menstrual cycle	rsfMRI	–	Group comparison	Greater activity in right DLPFC during mid-luteal phase vs. late follicular phase During the late follicular phase, relative progesterone levels were positively associated with ALFF in right HIP, thalamus, precuneus, and left angular gyrus. No associations between estradiol and brain activation During the mid-luteal phase, estradiol was positively associated with bilateral DLPFC and superior medial PFC ALFF. Relative progesterone levels positively correlated with temporal cortex ALFF
Menting-Henry et al. (2022)	18 naturally cycling women, 16 androgenic OC users, 16 anti-androgenic OC users	25 (6)	Menstrual cycle, OC use	fMRI, structural MRI	Emotion recognition	Group comparison	No group differences in emotion recognition performance No group differences in AMY GMV Lower ALFF in left PCC was associated with higher recognition of disgust in anti-androgenic OC users Right superior parietal lobe ALFF during sadness recognition was positively associated in naturally cycling women and negatively associated in anti-androgenic OC users Left AMY and ACC connectivity was negatively associated for naturally cycling women during fear recognition Right AMY and left middle frontal gyrus connectivity during fear recognition was negatively associated in naturally cycling women and positively associated in anti-androgenic OC users
Noyan et al. (2022)	13 control subjects and 13 subjects with Schizophrenia	18–45	Menstrual cycle	rsfMRI	–	Repeated measures analysis	No differences in functional connectivity between groups or cycle phases Estradiol levels positively correlated with connectivity of auditory network in the left AMY during the early follicular phase in schizophrenia patients Progesterone levels positively correlated with connectivity between left FPN and precuneus during the early follicular phase Progesterone levels negatively correlated with connectivity between the ECN in right superior frontal gyrus. No associations between estradiol and functional connectivity

*Only studies since 2012 are included in the table.ACC, anterior cingulate cortex; ADC, apparent diffusion coefficient; ALFF, amplitude of low-frequency fluctuations; AMY, amygdala; BOLD, blood-oxygen-level-dependent; BrainAGE, Brain Age Gap Estimation; Cr, creatine; CSF, cerebrospinal fluid; DLPFC, dorsolateral prefrontal cortex; DMN, default mode network; EC, eigenvector centrality; ECN, executive control network; FA, fractional anisotropy; FFA, fusiform face area; fMRI, functional magnetic resonance imaging; FPN, frontoparietal network; GABA, gamma aminobutyric acid; GMV, gray matter volume; GnRHa, gonadotropin-releasing hormone agonist; HC, hormonal contraceptives; HIP, hippocampus; MDD, major depressive disorder; IPL, inferior parietal lobule; IUD, intrauterine device; MRI, magnetic resonance imaging; OC, oral contraceptives; PCC, posterior cingulate cortex; PET, positron emission tomography; PFC, prefrontal cortex; PHG, parahippocampal gyrus; PMDD, premenstrual dysphoric disorder; POST, post-menopausal; PRE, pre-menopausal; RSFC, resting-state functional connectivity; rsfMRI, resting-state functional magnetic resonance imaging; VMPFC, ventromedial prefrontal cortex; WMV, white matter volume.*

On the contrary, the volume of the basal ganglia and anterior cingulate cortex (ACC) is reduced during the late follicular phase, opposite the pattern observed for the hippocampus (Protopopescu et al., 2008; De Bondt et al., 2013a). In the mid-luteal phase, when estradiol levels are moderate and progesterone levels are high, ACC volume increased. The increase in ACC volume was inversely correlated with estradiol levels, and positively correlated with progesterone levels (De Bondt et al., 2013a; Pletzer et al., 2018). Other regions, including fusiform gyrus, insula, and some parts of the temporal and frontal cortices, also change in size across the menstrual cycle (Pletzer et al., 2018).

Functional MRI studies also provide evidence of differential activation patterns during the menstrual cycle. A recent systematic review of neuroimaging studies indicates increased prefrontal cortical activity during cognitive tasks during the mid-luteal phase (Dubol et al., 2021). There is mixed evidence for preferential ACC activation exhibits greater activation and functional connectivity during the early follicular (menstrual) phase and late follicular phase compared with the midluteal phase (Thimm et al., 2014), or in the midluteal compared to the late follicular phase (Diekhof and Ratnayake, 2016). Activity in hippocampus (Pletzer et al., 2019b) and insular cortex (Dubol et al., 2021) during cognitive activities tend to be greater during the follicular phase.

Additionally, resting state fMRI studies indicate that some regions within the Default Mode Network (DMN) are more connected in the early follicular phase, when estradiol and progesterone levels are low (Petersen et al., 2014; Weis et al., 2019). Instead, another study reported no impact of menstrual cycle phase on DMN connectivity but increased connectivity between basal ganglia and frontoparietal attention network in midluteal phase, when both progesterone and estradiol are high (Pletzer et al., 2016). Some studies showed higher functional connectivity between amygdala and cingulate cortex, and amygdala with middle frontal gyrus (Petersen et al., 2019), and between ACC and the executive control network during the follicular phase as compared to the luteal phase, whereas dorsolateral prefrontal cortex and sensorimotor cortex are more connected with hippocampus (Arélin et al., 2015), resulting in greater activity in response to stimuli (Dubol et al., 2021), during the luteal phase compared to the follicular phase. Another study has reported that the hippocampus has greater whole brain functional connectivity at rest during the mid-luteal phase (Hidalgo-Lopez et al., 2020). Finally, a study comparing all three phases of the menstrual cycle showed higher hippocampal activation during the pre-ovulatory phase (e.g., higher estradiol) and higher fronto-striatal activation during the luteal cycle phase (e.g., higher progesterone) (Pletzer et al., 2019b). However, other studies comparing the three phases of the menstrual cycle did not confirm these associations (Hjelmervik et al., 2014; De Bondt et al., 2015b). Additionally, a study comparing the early follicular and mid-luteal phases found increased connectivity between angular gyrus and DMN, and between ACC with executive control network (ECN), during the follicular phase as compared to the mid-luteal phase (Petersen et al., 2014). A smaller study comparing mid-follicular and late luteal phases found no functional connectivity differences between menstrual phases (Syan et al., 2017). However, progesterone levels were positively correlated with connectivity of frontoparietal network (FPN) and DMN regions during the late luteal phase.

Although PET studies of the menstrual cycle are scarce and limited by small sample sizes, they did provide evidence for bioenergetic changes over the menstrual cycle, and limited to no effects on neurotransmitter activity. On [^18^F]fluorodeoxyglucose (FDG) PET, cerebral glucose metabolism (CMRglc) was higher in thalamic, prefrontal, temporoparietal, and inferior temporal regions in the mid-follicular as compared to the luteal phase, whereas CMRglc in superior temporal, occipital, cerebellar, cingulate and anterior insular regions was higher in the luteal as compared to the follicular phase (Reiman et al., 1996). There were also no differences in overall brain glucose metabolic activity between the follicular and luteal phases as measured via FDG-PET (Rapkin et al., 2011). There is no evidence for changes in D2 dopamine receptor density during different phases of the menstrual cycle on [^11^C]raclopride PET (Nordström et al., 1998), or for differences in serotonin binding between men and women in the follicular phase on [^11^C]WAY-100635 PET (Stein et al., 2008). One study of [^11^C]vorozole PET found no differences in aromatase activity between midcycle and late luteal phases (Biegon et al., 2015). A double-blind, randomized, placebo-controlled study investigating the effects of a gonadotropin-releasing hormone agonist (GnRHa) used [^11^C]DASB PET to image serotonin transporter (SERT) binding during the follicular phase in naturally cycling women (Frokjaer et al., 2015). The researchers found that increased SERT binding in neocortex and lower estradiol levels in the GnRHa group was associated with depressive symptoms as compared to placebo (Frokjaer et al., 2015). Another study using [^11^C]DASB PET reported that lower serum levels of allopregnanolone, which typically occurs during the follicular phase, was associated with greater SERT binding in prefrontal cortex (PFC) (Sundström Poromaa et al., 2019). However, several studies did not perform follow-up scans during different menstrual cycle phases (Frokjaer et al., 2015; Sundström Poromaa et al., 2019), thus additional work is needed to elucidate the relationship between menstrual cycle effects on PET brain imaging.

Altogether, neuroimaging results indicate that hormonal changes during the menstrual cycle may impact widespread networks involved in memory, learning, attention, and emotion. It is possible that, as effects of ovarian hormones on synaptic activity are generally subtle, neuroimaging might be more sensitive to detecting these changes than cognitive tests. Since most fMRI studies show no links to cognitive performance despite detecting activational changes during the menstrual cycle, it’s been hypothesized that not all effects of ovarian hormones might immediately translate to changes in cognition (Pletzer et al., 2019b). It is also possible that the brain compensates for cycling variations in ovarian hormone levels, leaving cognitive performance broadly unchanged throughout the menstrual cycle. Further, recent reviews suggest that menstrual cycle-related changes in cognition may be smaller than those in affective function and mood (Sacher et al., 2013). It is well established that the risk of depression becomes higher in women than in men starting at puberty (McGuire et al., 2019), and midlife depression is a risk factor for AD in turn (Livingston et al., 2020). Whether links between menstruation and mood are predictors of cognitive vulnerability later in life is under investigation.

## Oral contraceptives

Hormonal contraceptives consist either of a synthetic progesterone (i.e., progestin), or a progestin and a synthetic estrogen (e.g., combined formulation). These exogenous hormones control ovulation by inhibition of follicular development, and suppression of the production of endogenous estradiol and progesterone (Taylor et al., 2021). Hormonal contraceptives have various routes of administration, including oral, transdermal, intrauterine, and transvaginal. The most common form of birth control is by means of oral contraceptives (OC), which are used by over 85% of women in the United States (Taylor et al., 2021). Most OC formulations contain 21 active pills followed by seven placebo pills, which do not halt menstruation. Placebo pills are placeholders meant to help you stay on track by taking one pill every day until the next month starts. Some formulations have longer or shorter pill phases. Other formulations contain 28 active (monophasic) pills, which halt menstruation. Most OC contain ethinylestradiol, a potent form of estradiol, and synthetic progestins with different hormone derivatives. As a result, pills can either be androgenic or anti-androgenic (Pletzer et al., 2019a; Taylor et al., 2021).

Given the effects of ovarian hormones on brain structure and function, examination of the effects of OCs on cognitive aging and AD risk provides important information for preventative efforts. Nonetheless, few studies have investigated whether OC use influences cognition. Most of these studies were conducted on young adult women, while a handful examined associations between OC use in young adulthood and midlife, and future risk of cognitive decline in older age. While some studies report no differences in cognitive performance between young adult women with natural cycles and OC users (Lisofsky et al., 2016), others suggest that OC therapy supports verbal memory (Warren et al., 2014; Beltz et al., 2015) but not verbal fluency (Griksiene et al., 2018). Users of pills with androgenic progestins may also show increased spatial ability (Griksiene et al., 2018). For the long-term, some studies report higher performance on cognitive testing (Egan and Gleason, 2012; Karim et al., 2016) or a reduced risk of cognitive impairment (Li et al., 2016; Song X. et al., 2020) in midlife women taking OC. One study reported an almost 50% lower risk of cognitive impairment in women aged 60 or older who had used birth control as compared to never-users (Li et al., 2016). The remaining studies report no associations between OC use and cognitive performance (Ryan et al., 2009; Tierney et al., 2013), cognitive decline (McLay et al., 2003), or dementia incidence (Najar et al., 2020). Inconsistent findings may be a result of discrepancies in several factors including the age of initiation, OC formulations, dosage and duration of use (Taylor et al., 2021).

Neuroimaging studies of OC use are summarized in Table 1. Generally, structural MRI studies of young adult women indicate that OC users have larger regional gray matter (GM) volumes than natural cycling women, chiefly in frontal, temporal and anterior cingulate cortices, as well as hippocampus, parahippocampal gyrus, and cerebellum (Pletzer et al., 2010, 2015; De Bondt et al., 2013a; Figure 1). Limited data from longitudinal studies suggest that frontal and ACC volumes may be larger during the active phase compared with the placebo phase, during which no hormones are given (Pletzer et al., 2010; De Bondt et al., 2013a). Another study observed larger hippocampal volume with longer duration of OC treatment in young adult women, although the associations were mild (Pletzer et al., 2019a). In a recent MRI study of midlife women at risk for AD, OC users exhibited greater GM volume in medial temporal lobe, precuneus, fusiform gyrus, parietal and frontal cortex as compared to never-users (Schelbaum et al., 2021), which is in line with findings in younger women (Pletzer and Kerschbaum, 2014). However, other studies reported reduced GM volume of amygdala, parahippocampal gyrus, hypothalamus, pituitary gland, posterior cingulate cortex and orbitofrontal cortex of OC users compared to non-users (Petersen et al., 2015; Lisofsky et al., 2016; Chen et al., 2021). When comparing the follicular phase of naturally cycling women with the inactive phase of androgenic progestins or antiandrogenic pills, OC users had lower GM volume in cingulate gyrus and bilateral culmen, although these effects did not survive correction for multiple comparisons (De Bondt et al., 2016). The OC formulation also seems to matter, as women taking pills with androgenic progestins demonstrated smaller frontal volume and lower face recognition performance as compared to non-users, whereas those taking antiandrogenic pills had larger parahippocampal and fusiform volumes and better cognitive scores (Pletzer et al., 2015).

Most fMRI studies report an overall lack of performance differences between OC users and naturally cycling women during processing tasks (Brønnick et al., 2020; Taylor et al., 2021), although some studies indicate reduced frontoparietal activation in OC users compared with non-users in the follicular phase, and greater medial PFC and inferior parietal activation in OC users compared with non-users in the midluteal phase (Pletzer et al., 2014). Resting state fMRI studies have also produced mixed results, as some studies report no differences between women using OC and naturally cycling women (De Bondt et al., 2015b), whereas others report mixed effects (Brønnick et al., 2020; Taylor et al., 2021). On Diffusion Tensor Imaging (DTI), young OC users exhibited higher mean diffusivity (MD) when compared to naturally cycling women in the luteal phase (De Bondt et al., 2013b). Another study of 45–80 year old women reported reductions in fractional anisotropy (FA) with duration and age at onset of OC use (Nabulsi et al., 2020), while a separate study reported higher FA in younger OC users compared to naturally cycling women (Sharma et al., 2020).

Overall, research concerning OC effects on cognitive aging is just emerging. Although samples are small and differences between OC formulations were not reported in most studies, there is some indication that exogenous hormones influence brain volumes among young adult OC users, and may play a role in verbal functions, consistent with research on the menstrual cycle. Future systematic work is needed to better elucidate androgenic vs. anti-androgenic OC effects on cognitive health, and to probe between OC use pre-menopause and cognition post-menopause. Given the widespread use of OC, this work carries significant implications.

## Pregnancy

Pregnancy induces significant changes in endogenous estrogen levels, with reported effects on brain structure and function (de Lange et al., 2020). High levels of estradiol observed during pregnancy may lend neuroprotective support due to cumulative estrogen exposure (Deems and Leuner, 2020). However, the neurological impact of pregnancy is multifaceted and the biological mechanisms impacting cognitive aging remain to be elucidated. On one hand, compared to women who have never been pregnant, the levels of circulating estrogen are lower in women who have experienced pregnancy, a difference which extends into menopause (Bernstein et al., 1985). On the other hand, brain sensitivity to estrogen is increased in pre-clinical models of pregnancy, as evidenced by increased numbers of ERα positive cells in parous rats compared to nulliparous rats (Byrnes et al., 2009). Reports also suggest these effects may be evident in the human brain, as parity has been associated with increased responsiveness to estrogen in older aged women (de Lange et al., 2019).

Nonetheless, the vast majority of studies have focused on the short-term effects of pregnancy and postpartum on brain structure, function, and cognition, with the longest follow-ups conducted at 2–6 years postpartum (Brunton and Russell, 2008; Barth and de Lange, 2020). Studies investigating the long-term effects of pregnancy and childbearing on cognitive aging and AD risk are scant, as summarized below.

There is some evidence for a positive effect of pregnancy on cognitive aging. Several studies have reported that midlife women who had experienced pregnancy exhibited better cognitive performance in verbal and visual memory performance (Henderson et al., 2003; Ning et al., 2020), and another reported lower AD risk in later life (Fox et al., 2018). Studies examining gravidity (total number of pregnancies including stillbirth, miscarriage, and/or abortion) have reported a reduced risk of AD in elderly women who had spent more cumulative months pregnant and breastfeeding throughout their life (Fox et al., 2013, 2018). Another study supported these findings in reporting protection against AD dementia with longer breastfeeding duration (Heys et al., 2011). During lactation, estrogen levels are lower, and thus there are likely other factors contributing to these associations.

However, other studies report detrimental effects of pregnancy on cognitive aging. Compared to nulliparous women, parous women had greater cognitive decline on Mini-Mental State Examination (MMSE) scores (McLay et al., 2003), increased AD risk (Colucci et al., 2006) and AD onset at a younger age (Ptok et al., 2002), which may be limited to non-carriers of the ApoE4 gene (Corbo et al., 2007). A post-mortem study reported no clear associations between cognition and parity, though parity was associated with higher levels of AD-related neuropathology (Beeri et al., 2009).

Other studies have reported null associations between parity and cognitive performance or dementia risk (Ptok et al., 2002; Corbo et al., 2007; Ryan et al., 2009; Bae et al., 2020). In the Rancho Bernardo Study, 1,025 women between the ages of 44–99 who were followed over time showed no long-term effect on cognitive performance in relation to their prior pregnancies (Ilango et al., 2019).

Discrepancies may be in part due to how studies define parity. Studies defining parity as the number of childbirths or time spent pregnant more commonly report associations with cognition as compared to studies defining parity as parous vs. nulliparous. The number of children may play an important role, studies report having 1–4 children provides neuroprotection in women (Heys et al., 2011; Ning et al., 2020; Song X. et al., 2020), having 5 or more children, or grand multiparity, has been linked to negative effects as measured by cognitive performance or dementia risk (Rasgon et al., 2005a; Bae et al., 2020; Song X. et al., 2020).

While neuroimaging results are also mixed, MRI studies generally report positive effects of pregnancy and parity on structural brain aging (Figure 1). Two large studies reported that in comparison to nulliparous women, parous women, especially with a higher number of childbirths, exhibited less apparent brain aging as predicted via MRI-based machine learning models (de Lange et al., 2020; Ning et al., 2020). A recent volumetric MRI study of cognitively normal midlife individuals at risk for AD reported positive associations between number of children (between 2 and 5) and larger GM volume in frontal and temporal regions in women, whereas no associations were observed among men (Schelbaum et al., 2021). While there was no direct association between cognitive performance and number of children, there was a positive association between temporal cortex GMV with memory and global cognition performance, which suggests a mediation effect of pregnancy on cognition (Schelbaum et al., 2021).

Overall, studies investigating the associations between pregnancy and later life cognition are limited by small samples, heterogeneity of cognitive assessments and diagnostic criteria, possible inclusion of non-biological children, and different exposure variables. Pregnancy-related factors, including age at first birth, breastfeeding, or complications such as gestational diabetes or pre-eclampsia, have rarely been considered yet may have significant contributions. Later life cognitive testing or dementia diagnosis may also contribute to contrasting results, as the effects of pregnancy are likely more apparent closer to the time of childbirth than many years later after cumulative experiences have affected the brain.

## The menopause transition

Menopause represents the permanent cessation of ovulation and menstrual cycles, which is defined retrospectively, after 12 months of amenorrhea without obvious pathologic cause (Harlow et al., 2012). Hormonally, menopause is characterized by drastic reductions in estradiol and progesterone levels and elevated levels of gonadotropins follicle-stimulating hormone (FSH) and luteinizing hormone (LH) (Santoro, 2005). Menopause occurs either as the result of a natural midlife aging process (spontaneous menopause) or iatrogenically, via surgical or pharmacological intervention (induced menopause). In most cases, induced menopause results from bilateral oophorectomy or salpingo-oophorectomy, which lead to an abrupt cessation of ovarian estrogen production. Hysterectomy without oophorectomy can reduce ovarian estrogen production by disturbing blood flow to the ovaries, thus indirectly influencing the onset of menopause (Jett et al., 2022). Endocrine therapy for cancer and radiation therapies can also damage the ovaries and precipitate menopause (Jett et al., 2022). The reduction in ovarian hormones, particularly estradiol, is thought to elicit vasomotor (e.g., hot flashes) and urogenital (e.g., vaginal dryness) symptoms, while also increasing risk for cardiovascular disease and osteoporosis (Harlow et al., 2012), as well as neurological and psychiatric disorders including depression, anxiety, and dementia (Monteleone et al., 2018).

The average age at spontaneous menopause in industrialized countries is 49–51 years (Monteleone et al., 2018). Therefore, women live at least a third of their lives in a hypogonadal state, and that number increases to up to half for women with induced menopause (Monteleone et al., 2018). Recent evidence that AD starts in midlife (Sperling et al., 2013), thus proximate to the menopause transition, has highlighted a previously overlooked connection between menopause and AD risk. Currently, menopause is the most widely investigated female-specific risk factor for AD (Rahman et al., 2020). Estrogen withdrawal during menopause has been linked to accelerated brain cellular aging, possibly increasing risk of neurodegenerative events and AD later in life (Wang et al., 2020b; Mosconi et al., 2021).

Spontaneous menopause is a normal physiological event without long-term adverse effects for the majority of women (Monteleone et al., 2018). However, as high as 80% of women are vulnerable to the neurological shifts that can occur during this transition (Brinton et al., 2015), experiencing not only vasomotor symptoms such as hot flashes, but also “brain fog” and cognitive complaints. While the term “brain fog” is not a medically accepted entity, it reflects the common self-reported awareness of a decline in memory, attention and concentration during the menopause transition (Gold et al., 2000). While statistics on this vary, over 60% of women report changes in their ability to think clearly, concentrate, remember, or make use of new information during the menopause transition (Greendale et al., 2020). Most women experience a 15–20% increase in forgetfulness during perimenopause relative to pre-menopausal levels (Gold et al., 2000).

Nonetheless, whether menopause-related cognitive complaints can be confirmed objectively is a topic of debate (Mitchell and Woods, 2011; Weber et al., 2012). The first evidence for associations between menopause and memory decline stemmed from studies of oophorectomy, which reported an almost doubled long-term risk of dementia in oophorectomized women (Rocca et al., 2007, 2014; Phung et al., 2010; Bove et al., 2014). Dementia risk is generally highest following bilateral oophorectomy, intermediate with unilateral oophorectomy, and lowest but significant following hysterectomy without oophorectomy (Yaffe et al., 1998; Hogervorst et al., 2000; LeBlanc et al., 2001; Rocca et al., 2007; Phung et al., 2010; Bove et al., 2014; Gilsanz et al., 2019). For example, The Mayo Clinic Cohort Study of Oophorectomy (MCSO) observed an 84% higher risk of dementia for women who underwent unilateral oophorectomy with or without hysterectomy before age 42 years, and a 70% to double higher risk in women who underwent bilateral oophorectomy before the onset of natural menopause (Rocca et al., 2012). Phung et al. (2010) reported a 38% higher risk of dementia before the age of 50 for hysterectomy alone [RR = 1.38, 95% confidence interval (CI) = 1.07–1.78], and over double the risk with unilateral oophorectomy (RR = 2.10, 95% CI = 1.28–3.45) and bilateral oophorectomy (RR = 2.33, 95% CI = 1.44–3.77) (Phung et al., 2010). Dementia risk increases with younger age at the time of surgery (Rocca et al., 2008; Phung et al., 2010), which has also been associated with an increased burden of AD neuropathology at post-mortem (Bove et al., 2014; Agca et al., 2020). Surgical menopause may also have more severe consequences on cognitive function, including lower performance in verbal learning, visual memory (Rocca et al., 2007), and delayed word recall tasks (Zhou et al., 2011). Decline in short-term verbal memory was more severe in women who had greater than 50% decline in serum estradiol levels following surgery (Nappi et al., 1999; Farrag et al., 2002).

Overall, studies including surgical and spontaneous menopause cases indicate measurable, yet modest declines in verbal episodic memory on delayed recall tests, or lack of improvement in verbal memory and processing speed with repeated testing (Fuh et al., 2006; Greendale et al., 2009, 2011; Bromberger et al., 2010; Berent-Spillson et al., 2012; Epperson et al., 2013; Weber et al., 2013). In some studies, peri-menopausal women exhibited declines in working memory and complex attention rather than verbal episodic learning or memory (Weber et al., 2012), suggesting that operations demanding higher cognitive effort contribute to women’s perception of cognitive difficulties.

Some studies indicate that cognitive changes are possibly transient, as evidenced by longitudinal reports suggesting that they are mostly present at the peri-menopausal and early post-menopausal stages, with a rebound to almost pre-menopausal levels after menopause (Greendale et al., 2009; Weber et al., 2013). In the Study of Women Across the Nation (SWAN), over 2,300 midlife women followed for 4 years showed a decrease in verbal memory and processing speed in perimenopause compared to their pre-menopausal scores (Greendale et al., 2009). These declines resolved post-menopause, when cognitive performance returned to pre-menopausal levels, or closer to baseline (Greendale et al., 2009). In the Rochester Investigation of Cognition Across Menopause, peri-menopausal and early post-menopausal women had lower verbal memory, attention, and working memory scores, which improved in late postmenopause (Weber et al., 2013). However, other studies report conflicting results of reduced memory still in postmenopause (Epperson et al., 2013). While cognitive effects are for the most part independent of non-cognitive menopausal symptoms such as anxiety and disturbed sleep (Greendale et al., 2010), frequent hot flashes and a negative mood have been linked with more severe cognitive disturbances (Maki et al., 2008; Drogos et al., 2013).

Importantly, memory declines during perimenopause and early postmenopause ranged from subtle to moderate, and remained within normal limits for age and education in most studies (Maki and Henderson, 2016). Moreover, women maintain an advantage in verbal memory as compared to age-controlled men regardless of menopausal status (Rentz et al., 2017), which strongly argue for development of gender-specific tests that also take account women’s reproductive stage. Generally, cognitive complaints during menopause are unlikely to result in objectively measured impairments, thus often falling under the diagnostic category of subjective cognitive decline (SCD). Current evidence suggests that people ages 65 and older experiencing SCD may be at higher risk for MCI and dementia (Jessen et al., 2014), especially women (Pérès et al., 2011).

Although neuroimaging research of menopause is scant, and the majority of studies has been carried out in women who had already transitioned through the menopause, recent translational neuroimaging studies corroborate animal findings by showing associations between menopause and biomarker indicators of AD risk in midlife women (Rahman et al., 2019; Jett et al., 2022). Neuroimaging studies of menopause status are summarized in Table 2.

**TABLE 2:** Observational studies of menopause status and menopausal hormone therapy (MHT) use on neuroimaging outcomes.

Study	Exposure	Participants	Age, years	Imaging modality	Cognitive measures	Study design	Main findings
Eberling et al. (2000)	MHT use	8 MHT users, 5 non-users, 13 AD patients	74 (8)	FDG-PET	–	Group comparison	Higher CMRglc in MHT users vs. AD patients No CMRglc difference between non-users and AD patients
Maki and Resnick (2000)	MHT use	12 MHT users, 16 non-users	55+	^15^O-water PET	Verbal Memory, Visual Memory, Psychomotor Speed	2 year longitudinal study	Greater increases in relative CBF in MTL, insula, cerebellum, frontal, and temporal cortex in MHT users vs. non-users Greater CBF increase in ACC in non-users vs. MHT users Greater CBF increases in insula, HIP, and temporal cortex during verbal memory task in MHT users vs. non-users Better performance on neuropsychological memory tasks in MHT users vs. non-users
Słopień et al. (2003)	Menopause status, MHT use	10 PRE, 20 POST	PRE: 33 (13), POST: 49 (5)	SPECT	–	Group comparison; follow-up SPECT on 10 women with low CBF who were put on MHT	Lower CBF in POST vs. PRE Ventricular CBF improved after 1 year of MHT use
Erickson et al. (2005)	MHT use	16 current MHT users, 14 past MHT users, 13 non-users (all POST)	57–79	Structural MRI	–	Group comparison	Larger GMV in frontal, temporal, and parietal cortex in all MHT users vs. non-users MHT duration positively associated with GMV in PFC, parietal and temporal cortex Larger WMV in medial temporal lobe in all MHT users vs. non-users
Rasgon et al. (2005b)	MHT use	11 MHT users, 9 non-users	50–84	FDG-PET	MMSE, Buschke-Fuld total recall, Delayed paragraph recall, Benton visual errors	2 year longitudinal study	PCC CMRglc decline in non-users vs no decline in MHT users No differences in cognitive performance
Boccardi et al. (2006)	MHT use	16 current tE2 users, 7 past MHT users, 17 non-users (all POST)	50+	Structural MRI	–	Group comparison	Larger global GMV in tE2 users vs. non-users, with peaks in cerebellum, middle temporal and inferior frontal gyri Larger GMV in cerebellum, middle temporal and inferior frontal gyri of past users vs. non-users
Gleason et al. (2006)	MHT use	4 opposed E2 users, 10 opposed CEE users, 9 non-users	59 (5)	fMRI	Auditory Verbal Learning Test; fMRI: line drawing task	Group comparison	Greater HIP activation in MHT users vs. non-users Estradiol users exhibited the best verbal memory performance, non-users intermediate, and CEE users the worst performance
Greenberg et al. (2006)	MHT use	41 current MHT users, 51 non-users (all POST)	60+	Structural MRI	MMSE, extensive neuropsychological evaluation including verbal fluency, verbal memory, visual memory	Group comparison	Smaller GMV and larger non-ventricular CSF volume in MHT users vs. non-users No differences in cognitive performance
Low et al. (2006)	MHT use	64 current MHT users, 69 past MHT users, 80 non-users (all POST)	60–64	Structural MRI	Verbal intelligence	Group comparison	No differences in GMV Past MHT users exhibited the highest verbal intelligence, non-users intermediate, and current MHT users the lowest
Lord et al. (2008)	MHT use	16 current unopposed estrogen users, 10 past MHT users, 15 non-users (all POST)	50–74	Structural MRI	–	Group comparison	Larger right HIP GMV in current ET users vs. past users and non-users Negative association between HIP GMV and ET duration in current users but not past users No group differences in AMY GMV
Berent-Spillson et al. (2010)	MHT use	13 current MHT users vs. 24 past MHT users vs. 18 non-users (all POST)	60+	fMRI	Visual Delayed Matching to Sample task	Group comparison	No group difference on visual memory performance Greater activation in HIP, insula, PCC, ACC, parietal and frontal cortex for MHT users vs. non-users Greater activation in HIP, insula, frontal and parietal cortex in EPT users vs. non-users Greater activation in left parietal cortex and PHG for EPT users vs. ET users Greater activation in right parietal and frontal cortex in ET vs. EPT users Greater activation in right PFC for past MHT users vs. current users
Maki et al. (2011)	MHT use	13 MHT users vs. 12 non-users (all POST)	56–67	fMRI	Verbal memory: CVLT-II, EBMT, Unrelated Word List, Wechsler Memory Scale-III Faces subtest	Group comparison	POST women who had initiated MHT during PERI exhibited greater activation in left HIP but lower activity in bilateral PHG during recognition and match conditions of verbal memory tasks vs. non-users Better verbal recognition task performance in MHT users vs. non-users
Silverman et al. (2011)	MHT use	53 POST	49–69	FDG-PET	Auditory Consonant Trigrams, Benton Visual Retention Test, Boston Naming Test, Trail Making Test Rey-Osterrieth Complex Figure Test, Logical Memory	Baseline results from 2 year prospective randomized study	Higher CMRglc in left parietotemporal cortex and right temporal gyrus in 17β-estradiol users vs. CEE users 17β-estradiol users scored significantly higher on verbal memory performance vs. CEE users Positive association between verbal memory performance and CMRglc in Wernicke’s and auditory association areas in E2 users Positive association between verbal memory performance and CMRglc in right superior frontal gyrus in CEE users Higher CMRglc in bilateral temporal cortex and frontal cortex in unopposed MHT users vs. opposed MHT users
Shafir et al. (2012)	MHT use	15 CEE users, 20 CEE + MPA, 17 non-users (all POST)	60–81	fMRI	Emotional processing	Group comparison	Lower activation in left medial frontal gyrus and anterior cingulate during positive stimuli processing in ET users vs. non-users Lower activation in right posterior insula during positive stimuli processing in EPT users vs. non-users Greater activation in right entorhinal cortex during negative stimuli processing in ET users vs. non-users No brain activation differences between ET and EPT users Greater activation in right HIP during positive stimuli processing in all current MHT users vs. all past users
(Ryan et al., 2014)	MHT use	62 current users, 60 past users, 173 non-users (all POST)	68–75	Structural MRI	–	Group comparison	Smaller total GMV in current users vs. past and non-users No differences in HIP, corpus callosum, or white matter lesion volume
Stein et al. (2014)	Menopause status	16 PRE, 28 POST	PRE: 20–35; POST: 50–65	^11^C WAY-100635 PET	–	Group comparison	Negative associations between progesterone levels and 5-HT1A serotonin binding in ACC in POST but not PRE Negative associations between DHEAS levels and 5-HT1A binding in AMY in POST but not PRE No association between estradiol levels and 5-HT1A binding in PRE or POST
Jovanovic et al. (2015)	MHT use	10 POST (all surgical)	40–65	^11^C-MADAM PET	Trail Making Tasks A + B, “Reading the mind of the eyes” (social cog. Test), controlled oral word association test (FAS and categories)	6 month longitudinal study	Significant decrease in 5-HTT serotonin binding in frontal, parietal, occipital, temporal cortex, MTL and basal ganglia during MHT use vs. baseline Women with tE2 + testosterone treatment performed better on verbal fluency tasks vs. baseline
Thurston et al. (2015)	Hot flashes number and severity	3 PERI, 17 POST	40–60	rsfMRI	–	Association study	Positive association between physiologically-monitored hot flashes and DMN connectivity
Jacobs et al. (2016)	Menopause status	32 PRE, 29 PERI, 31 POST	45–55	fMRI and rsfMRI	Digit span, Controlled Oral Word Association Test, American National Adult Reading Test, 12-item Face Name Associative Memory Exam, 6-trial Selective Reminding Test	Group comparison	Decreased HIP activation but greater HIP connectivity during verbal processing for POST vs. PRE and PERI HIP activity positively correlated and HIP connectivity, and negatively correlated with declining estradiol
Thurston et al. (2016)	Hot flashes number and severity	3 PERI, 16 POST	40–60	Structural MRI (WMHV)	–	Association study	Positive association between physiologically-monitored night sweats and WMHV
Vega et al. (2016)	Menopause	31 POST	50–60	rsfMRI	Cognitive complaints	Association study	Positive association between cognitive complaints and ECN nodes, but not DMN nodes
Berent-Spillson et al. (2017)	Menopause status	15 PRE, 11 PERI, 28 POST	42–61	fMRI	Cognitive control of emotion processing	Group comparison	No group differences. On *post hoc* analysis, PERI group activated right TPO junction, while POST group activated PFC, PCC and TPO junction during emotion processing
Braden et al. (2017)	MHT use	32 current MHT users, 41 past users, and 21 non-users (all POST)	73–91	Structural MRI	CVLT-II or Rey Auditory Verbal Learning Test	Group comparison	No differences in HIP volume in MHT users vs. non-users HIP volume correlated with verbal memory for non-users but not for MHT users
Jacobs et al. (2017)	Menopause status	26 PRE, 25 PERI, 20 POST	46–53	fMRI and rsfMRI	Verbal working memory	Group comparison	During verbal working memory task, increased DLPFC activation, but attenuated HIP deactivation across menopausal transition, which correlated with declining estradiol Greater DLPFC-HIP connectivity for POST vs. PRE, which correlated with verbal working memory for POST women only
Mosconi et al. (2017)	Menopause status	15 PRE, 13 PERI, 14 POST	40–60	Structural MRI, PiB-PET, FDG-PET	Digit symbol substitution, paired associates delayed recall, paragraph delayed recall, designs, object naming, WAIS vocabulary	Group comparison	Lower GMV and WMV in frontal cortex of PERI and POST vs. PRE Lower CMRglc in PCC, temporal and parietal cortex of PERI and POST vs. PRE Higher amyloid burden in PERI and POST vs. PRE ApoE4 + POST exhibited greatest amyloid burden in frontal cortex of all groups
Berent-Spillson et al. (2018)	MHT use	38 long-term MHT users vs, 19 non-users (all POST)	60+	fMRI	Verbal processing	Group comparison	Greater frontal activation during verbal processing in MHT users vs. non-users Longer response times during verbal discrimination and recall tasks in MHT users vs. non-users
Kim et al. (2018)	Menopause status	20 PRE, 20 POST	PRE: 40 (9) vs. POST: 56 (2)	Structural MRI	–	Group comparison	Reduced GMV in SMA, frontal and temporal regions of POST vs. PRE GMV differences correlated with estradiol levels
Mosconi et al. (2018a)	Menopause status	15 PRE, 14 PERI, 14 POST	40–60	FDG-PET and plasma COX	Digit symbol substitution, paired associates delayed recall, paragraph delayed recall, designs, object naming, WAIS vocabulary	Group comparison	Lower CMRglc in PCC, frontal, parietal and temporal cortex of POST vs. PRE Lower CMRGlc in PCC, temporal and frontal cortex in POST vs. PERI Lower CMRglc in PCC, temporal and parietal cortex of PERI vs. PRE Reduced COX activity in PERI and POST vs. PRE Lower verbal memory scores in POST vs. PRE
Mosconi et al. (2018b)	Menopause status	15 PRE, 14 PERI, 12 POST	40–60	Structural MRI, PiB- and FDG-PET	Digit symbol substitution, paired associates delayed recall, paragraph delayed recall, designs, object naming, WAIS vocabulary	Group comparison over 3 years	Greater rates of amyloid accumulation in frontal cortex and PCC in POST vs. PRE Greater rates of amyloid accumulation in frontal cortex in PERI vs. PRE Greater rates of CMRglc and HIP GMV decline in frontal cortex in POST vs. PRE and PERI Higher rates of decline in higher-order processing in POST vs. PRE and PERI
Zhang et al. (2018)	Menopause status	44 PRE, 43 POST	45–50	rsfMRI	Attention Network Task, Stroop Test, One-back working memory task	Group comparison	Higher DC in AMY, and lower DC in middle occipital gyrus in POST vs. PRE In POST group, AMY-PFC connectivity was positively associated with executive function accuracy In POST group, decreased connectivity between middle occipital gyrus and inferior parietal gyrus associated with lower working memory scores Longer reaction times and lower accuracy on cognitive tests for POST vs. PRE
Seitz et al. (2019)	Menopause status	33 PRE, 29 PERI, 32 POST	46–53	Structural MRI	Digit span, Controlled Oral Association Test for verbal fluency of letters and categories, American National Adult Reading Test, Buschke Selective Reminding Task, Face Name Associative Memory Task	Group comparison	Positive associations between GMV in ACC with HIP, inferior parietal cortex, and DLPFC in POST vs. no associations in PERI In POST group, women exhibiting higher associations between ACC and HIP performed better on Buschke memory task vs. those exhibiting lower associations
Nabulsi et al. (2020)	MHT use	3,106 MHT users vs. 5,195 non-users (PRE and POST)	45–80	DTI	–	Group comparison	Slower decline in WM fiber coherence loss with age in MHT users vs. non-users WM preservation in ET users vs. EPT users
Rahman et al. (2020)	Menopause status, MHT use	16 PRE, 27 PERI, 42 POST	40–65	Structural MRI, PiB-PET, FDG-PET	Digit symbol substitution, paired associates delayed recall, paragraph delayed recall, designs, object naming, WAIS vocabulary	Group comparison	Higher CMRglc in frontal and parietal cortex, and lower amyloid burden in orbitofrontal gyrus in MHT users vs. non-users
Boyle et al. (2021)	MHT use	562 POST	71–94	Structural MRI	Modified MMSE, Benton Visual Retention Test, Digit Symbol Substitution Test	Group comparison	Larger total GMV in CEE MHT users vs. non-users
He et al. (2021)	Menopause status	32 PRE, 25 PERI	45–55	rsfMRI	MMSE	Group comparison	Increased ReHo in lingual gyrus and lower ReHo in superior frontal gyrus of PERI vs. PRE In PERI group, ReHo in frontal areas positively correlated with MMSE score
Liu et al. (2021)	Menopause status	25 PRE, 25 PERI	45–55	rsfMRI	Stroop test	Group comparison	Increased ALFF in gyrus rectus and decreased ALFF in inferior frontal gyrus, insula and superior temporal gyrus of PERI vs. PRE Lower GMV in gyrus rectus and superior temporal gyrus of PERI vs. PRE Slower reaction rates in PERI vs. PRE
Mosconi et al. (2021)	Menopause status	30 PRE, 57 PERI, 74 POST	40–65	Structural MRI, ^31^P-MRS, PiB-PET, FDG-PET	Memory (immediate and delayed recall of a paragraph and paired associates), higher-order processing (block design tests), and language (object naming)	Group comparison, including 2-year longitudinal component	POST group exhibited lower GMV and higher ATP/PCr in temporal vs. PRE; lower WMV and CMRGlc in parietal and temporal vs. PRE and PERI; higher CBF in frontal, temporal, and parietal cortex vs. PERI PERI group exhibited lower GMV in precuneus and fusiform vs. POST; lower CMRglc in temporal cortex vs. PRE ApoE4 + POST and PERI exhibited greater amyloid burden vs. other groups POST group exhibited GMV increase in precuneus and stable WM and CMRglc measures at 2-year follow-up
Schelbaum et al. (2021)	Menopause status, MHT use	15 PRE, 35 PERI, 49 POST	40–65	Structural MRI	Rey Auditory Verbal Learning Test and Wechsler Memory Scale logical memory delayed recall tests, executive function (Trail Making Test B and F-A-S), and language (object naming) tests	Group comparison and associations	Lower GMV in frontal and temporal cortex of POST and PERI vs. PRE Larger GMV in fusiform, frontal, and temporal cortex of MHT users vs. non-users
Wisch et al. (2021)	MHT use	70 MHT non-users, 16 MHT users	Non-users: 68 (7), users: 70 (8)	Structural MRI, Tau-PET, PiB-PET	Free and Cued Selective Reminding Test, Logical Memory IIa subtest (Wechsler Memory Scale), Digit Symbol Substitution Test, MMSE	Group comparison	Better cognitive performance in MHT users vs. non-users Lower tau burden in MHT users vs. non-users
Zhang S. et al. (2021)	Menopause status	54 PRE, 45 early POST	45–51	Structural MRI	Stroop Test, Two-back working memory task	Group comparison	Lower AMY GMV in POST vs. PRE Longer reaction rates and lower Two-back working memory scores in POST vs. PRE

*ACC, anterior cingulate cortex; AD, Alzheimer’s disease; ALFF, increased amplitude of low-frequency fluctuation; AMY, amygdala; ApoE, apolipoprotein E; ATP, adenosine triphosphate; CBF, cerebral blood flow; CEE, conjugated equine estrogen; CMRglc, cerebral metabolic rates of glucose; COX, cytochrome oxidase; CSF, cerebrospinal fluid; CVLT, California Verbal Learning Test; DLPFC, dorsolateral prefrontal cortex; DC, degree centrality; DMN, default mode network; ECN, executive control network; ET, estrogen therapy; EPT, estrogen + progesterone therapy; E2, estradiol; FA, fractional anisotropy; fMRI, functional MRI; GMV, gray matter volume; HIP, hippocampus; 5-HTT, serotonin transporter protein; MHT, menopausal hormone therapy; MMSE, Mini-Mental State Examination; MPA, medroxyprogesterone acetate; MRI, magnetic resonance imaging; MTL, medial temporal lobe; PCC, posterior cingulate cortex; PCr, phosphocreatine; PERI, peri-menopausal; PET, positron emission tomography; PFC, prefrontal cortex; PHG, parahippocampal gyrus; POST, post-menopausal; PRE, pre-menopausal; ReHo, Regional homogeneity; ROI, region of interest; rsfMRI, resting state fMRI; SMA, supplementary motor area; SPECT, single photon emission computed tomography; tE2, transdermal estradiol; WAIS, Wechsler Adult Intelligence Scale; WM, white matter; WMHV, white matter hyperintensity volume; WHV, white matter volume.*

Recent multi-modality neuroimaging investigations targeting women at different menopausal stages (pre-menopausal, peri-menopausal, and post-menopausal), all carrying risk factors for AD, such as ApoE4 genotype and a family history of late-onset AD, demonstrate emergence of AD endophenotypes in women of peri-menopausal age (Mosconi et al., 2017, 2018a,b, 2021; Rahman et al., 2020). AD endophenotypes included higher Aβ load, lower CMRglc, and lower GM and WM volume in brain regions vulnerable to AD, chiefly posterior cingulate, precuneus, medial temporal, parieto-temporal, and frontal cortices as compared to pre-menopausal women and to age-controlled men, independent of age and midlife health indicators (Mosconi et al., 2017, 2018a,b, 2021; Rahman et al., 2020; Figure 1). Biomarker abnormalities increased post-menopause (Mosconi et al., 2017, 2018a,b, 2021; Rahman et al., 2020). Additionally, peri-menopausal and post-menopausal women positive for ApoE4 genotype exhibited the highest Aβ burden (Mosconi et al., 2017, 2021), supporting the notion that ApoE4 genotype exacerbates AD-related brain changes in women with onset in the perimenopause (Riedel et al., 2016). While menopause effects on Aβ deposition were overall mild, the earlier onset and longer exposure to Aβ pathology could help account for the higher prevalence of AD in women.

**FIGURE 2:**
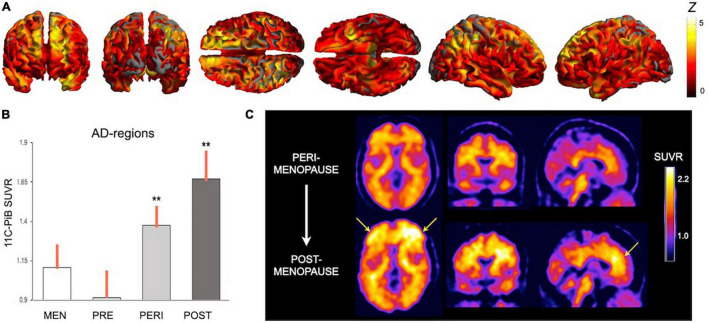
Effects of menopause on brain amyloid-beta deposition. Summary of Pittsburgh compound B PET (PiB-PET) studies showing menopause status effects on Aβ deposition: **(A)** Statistical parametric maps showing higher PiB uptake, a marker of Aβ load, in key brain regions for AD in a group of post-menopausal and peri-menopausal women vs. age-controlled men (Z scores > 2 correspond to p < 0.001). **(B)** In these regions, Aβ load was associated with menopausal status, e.g., was highest post-menopause, intermediate in peri-menopause, and lowest pre-menopause (**different from men at p < 0.001). **(C)** Aβ deposition is progressive during the menopause transition, as evidenced in a representative case who underwent PiB-PET at baseline, when she was peri-menopausal, and 3 years later, when she was post-menopausal. Images are adapted from data presented in **(A)** Mosconi et al. (2021), **(B)** Mosconi et al. (2017), and **(C)** Mosconi et al. (2018b). PiB, Pittsburgh compound B; SUVR, standardized uptake value ratio.

Longitudinal evaluations showed progressive AD biomarker abnormalities in the menopause transition, including chiefly declines in hippocampal and temporal lobe GM volumes, CMRglc declines in temporal regions and PCC, and increased Aβ deposition in frontal cortex (Mosconi et al., 2018b, 2021). Figure 2 provides an overview of menopause effects on Aβ deposition among midlife women.

Nonetheless, a follow-up study provided preliminary evidence for biomarker stabilization or recovery in late post-menopause (Mosconi et al., 2021). For example, GM volume declined during peri-menopausal and early post-menopausal stages (Mosconi et al., 2018b), but plateaued in temporal cortex, and showed a rebound in precuneus in late post-menopause (Mosconi et al., 2021). WM declines in major WM tracts and CMRglc in parieto-temporal areas also appeared to plateau in late post-menopause (Mosconi et al., 2021). Additionally, cerebral blood flow (CBF) measured by means of Arterial Spin Labeling (ASL) was higher in the post-menopausal group as compared to pre-menopausal controls and to age-controlled men, and so was the ratio of adenosine triphosphate (ATP) to phosphocreatine (PCr) levels measured by means of ^31^Phosphorus Magnetic Resonance Spectroscopy (31P-MRS), reflecting higher ATP synthesis (Mosconi et al., 2021). Importantly, cognitive performance was intact post-menopause, which correlated with GM volume and ATP levels (Mosconi et al., 2021). Biomarker “recovery” was, however, attenuated in peri-menopausal and post-menopausal ApoE4 carriers (Mosconi et al., 2021). Overall, while these findings need to be replicated in larger samples, in keeping with preclinical work (Wang et al., 2020c), they suggest presence of compensatory mechanisms that allow brain adaptation to the hypo-estrogenic post-menopausal state, at least in some women. Brain adaptation may also account for cognitive preservation and for the easing of menopausal symptoms observed in the late post-menopausal stage (Monteleone et al., 2018).

Other natural history studies indicate lower GM volume in post-menopausal as compared with pre-menopausal women in frontal and temporal regions, which positively corelated with estradiol levels (Kim et al., 2018). The type of menopause also seems to have an impact, as induced menopausal cases exhibited smaller medial temporal lobe volume as compared to spontaneous post-menopausal cases (Zeydan et al., 2019). Moreover, physiologically-monitored night sweats correlated with estrogen levels and white matter hyperintensities (Thurston et al., 2016). Albeit limited by the small samples and by the fact that cognition was not studied (Thurston et al., 2016), this study suggests a link between vasomotor symptoms and cerebral small vessel disease, a risk factor for later stroke and dementia (Debette and Markus, 2010).

fMRI studies also provide emerging evidence for menopause-related changes in brain activation during verbal tasks and emotion processing. In some studies, post-menopausal women showed the least hippocampal activation, in spite of increased hippocampal connectivity, during verbal processing (Jacobs et al., 2016, 2017). Post-menopausal women also exhibited increased dorsolateral prefrontal cortex activation during verbal working memory (Jacobs et al., 2017). There is also evidence that post-menopausal women exhibited increased activation of regions involved in cognitive control during emotion decision making, such as the PFC, posterior cingulate, and temporoparietal junction, but not in limbic system (Berent-Spillson et al., 2017). Finally, presence of subjective cognitive complaints was associated with increased connectivity of the prefrontal cortex (Vega et al., 2016) while physiologically-monitored hot flashes were linked to increased DMN connectivity (Thurston et al., 2015).

Overall, a growing literature indicates that ovarian steroid hormones, particularly declines in estradiol, reshape the landscape of the female brain during the menopause transition. Some aspects of memory, such as verbal memory, are negatively impacted by menopause, along with more variable declines in processing speed, attention, and verbal fluency. These effects are, however, mild and tend to resolve in the late post-menopausal stage, e.g., approximately 6 years after the last menstrual period. Novel neuroimaging data also suggest that negative effects of menopause on neurophysiology may be transient, with the exception of women at risk for AD, who exhibit preclinical AD endophenotypes already during perimenopause. However, given that all women experience menopause but only a fraction will develop dementia, more work is warranted to elucidate which protective mechanisms may offset the effects of menopause on AD risk. Population-based studies indicate that over 30% of all AD cases could potentially be prevented by addressing modifiable medical and lifestyle factors such as smoking, depression, obesity, diabetes, and lack of physical activity (Livingston et al., 2020). Many of these factors also impact the age of onset and severity of menopause (Monteleone et al., 2018). More studies are needed to examine the effects of lifestyle and medical comorbidities on the brain changes occurring during menopause in association with future AD risk.

It also remains unclear whether altered brain biomarkers and memory fluctuations during perimenopause are predictive of dementia in later life. There are also preliminary findings of links between white matter hyperintensities and vasomotor symptoms, and of menopause-related changes in cognitive processing during emotion identification and in resting state networks, which need further clarification. While available findings need to be replicated in larger samples with longitudinal follow-ups and with the use of AD biomarkers, the evidence so far indicates that window of opportunity for support of estrogen-based neuroplasticity is early in the endocrine aging process.

## Menopausal hormone therapy

Menopause hormonal therapy (MHT) includes oral and transdermal preparations thought to have systemic effects, and localized administrations (e.g., vaginal creams) that do not have systemic effects. Herein, we focus on systemic MHT. The treatment of choice for women with a uterus consists of combined (opposed) estrogens and progestins. The treatment of choice for women without a uterus is unopposed, or estrogen-only therapy. Estrogens can be estradiol or conjugated equine estrogens (CEE). Progestins vary in their hormone derivatives, as in hormonal contraceptives. The most commonly used are medroxyprogesterone acetate (MPA) and micronized progesterone.

There is a relatively large literature on MHT effects on brain and cognitive aging. In spite of this, results of whether MHT is viable for support of cognitive function and AD risk reduction are mixed. The hypothesis that MHT might protect against AD arose in large part from early observational studies and small clinical trials demonstrating a protective effect of MHT on cognitive function and AD risk among MHT users compared with never-users (Kawas et al., 1997; Zandi et al., 2002; Paganini-Hill et al., 2006; Sherwin, 2006; Whitmer et al., 2011), especially among younger, 50–59 year-old women (LeBlanc et al., 2001). Positive effects were particularly consistent with estrogen-only, or unopposed MHT for hysterectomized women (Sherwin and Phillips, 1990; Henderson et al., 2005; Rocca et al., 2007, 2010; Whitmer et al., 2011; Shao et al., 2012). Recent observational studies continue to provide conflicting results. For example, an analysis of health insurance claims from nearly 400,000 women reported a protective effect against AD and other neurodegenerative diseases with use of MHT (Kim et al., 2021). Compared with non-users, MHT users exhibited a 57% reduced risk of AD (Kim et al., 2021), with the greatest risk reduction for long-term MHT users (Kim et al., 2021). On the contrary, a population study in Finland of nearly 170,000 women reported that MHT use was associated with a 9–17% increased risk of AD, with higher risk for opposed MHT (Savolainen-Peltonen et al., 2019). In women younger than 60 at hormone therapy initiation, the increase in AD risk was mostly associated with MHT exposure over 10 years (Savolainen-Peltonen et al., 2019). However, ApoE4 status was not evaluated in this study. As Finland has a higher rate of ApoE4 carriers than most countries, with nearly 20% frequency, this is an important confounder as the effectiveness of MHT may be impacted by ApoE4 status (Depypere et al., 2016). Additionally, there is some evidence that oophorectomy before the natural age of menopause, but not after, is associated with an increased risk of AD (Rocca et al., 2007), which is mitigated by post-operative MHT (Sherwin and Phillips, 1990; Henderson et al., 2005; Rocca et al., 2007, 2010; Whitmer et al., 2011; Shao et al., 2012).

Randomized, placebo-controlled clinical trials of MHT for AD prevention have also provided conflicting results. The first large trial to test MHT for dementia prevention was the Women’s Health Initiative (WHI). The WHI included two studies, the WHI Estrogen-plus-Progestin Study, in which women with a uterus were randomly assigned to receive either combined MHT (Prempro) or a placebo; and the WHI Estrogen-Alone Study, in which women without a uterus were randomly assigned to receive either estrogen-alone therapy (Premarin) or a placebo. Cumulatively, the WHI showed some benefits related to use of MHT, including one-third fewer hip and vertebral fractures, and one-third lower risk of colorectal cancer relative to placebo (Rossouw et al., 2002; LaCroix et al., 2011). However, the trials were stopped prematurely as both MHT types were associated with an increased risk of coronary artery disease, stroke and blood clots (Rossouw et al., 2002; Anderson et al., 2004). Additionally, the Estrogen-plus-Progestin arm of the study initially showed an increased risk of cancer (Rossouw et al., 2002; Anderson et al., 2004), although subsequent analysis found no increase in risk (Anderson et al., 2012; Lobo, 2017).

The WHI included an additional arm, the WHI Memory Study (WHIMS), which examined the impact of MHT for dementia prevention among women ages 65 and older, thus in late post-menopause. These studies focused on oral CEEs in women with prior hysterectomy, and CEE/MPA in naturally post-menopausal women (Shumaker et al., 2003). Although AD was the *a priori* primary outcome of interest, all-cause dementia became the default primary outcome because of the lack of a sufficient number of AD cases at follow-up. In a sample of 2,947 post-menopausal women with prior hysterectomy, there was no evidence that CEE lowered the risk of all-cause dementia (Espeland et al., 2004; Shumaker et al., 2004). However, in a sample of 4,532 spontaneous post-menopausal women, CEE/MPA doubled the risk for all-cause dementia (Shumaker et al., 2003). Thus, the WHIMS study demonstrated no protective effects of unopposed MHT, and a substantial increase in dementia risk with opposed MHT among late post-menopausal women.

In terms of MHT effects on cognition, the WHIMS trial found that both opposed and unopposed therapy were associated with slightly worse mean scores in global cognitive function compared to placebo (Rapp et al., 2003; Espeland et al., 2004). These effects were observed within the first 3–4 years of the trial follow-up and remained fairly constant several years thereafter. The subsequent Women’s Health Initiative Study of Cognitive Aging (WHISCA) study examined whether MHT influenced domain-specific cognitive function at initial assessment, an average of 3 years after randomization to MHT or placebo, and after an additional ∼3 year of on-trial follow-up. Among the 2,304 participants, only small mean differences in cognitive test scores changes were noted (Resnick et al., 2006, 2009). Together, these findings suggest that if MHT use produces an initial decrement in at least some aspects of cognitive function, this decrement does not markedly widen or diminish thereafter. Notably, all the above studies involved post-menopausal women above age 65, thus possibly already harboring pre-existing cardiovascular or neurodegenerative conditions. As such, it may have been too late for MHT to prevent those conditions. These considerations, together with evidence from observational studies, has led to the understanding that the efficacy of MHT depends on the timing of initiation and the use of progestogens (LeBlanc et al., 2001; Manson et al., 2006; Maki, 2013; Bove et al., 2014).

However, the newer Early versus Late Intervention Trial with Estradiol (ELITE)-cog and Kronos Early Estrogen Prevention Study (KEEPS) trials have reported no beneficial or adverse effects of MHT on cognition among recently post-menopausal women within 6 years of the menopause diagnosis (Gleason et al., 2015; Henderson et al., 2016; Miller et al., 2019). Nonetheless, MHT reduced the progression of subclinical atherosclerosis when therapy was initiated soon after menopause (Hodis et al., 2016), which has been linked to a 30% reduced number of heart attacks and cardiac deaths (Salpeter et al., 2009).

To date, eight meta-analysis have examined the neuroprotective effects of MHT on AD risk (Yaffe et al., 1998; Hogervorst et al., 2000; LeBlanc et al., 2001; Lethaby et al., 2008; O’Brien et al., 2014; Song Y. J. et al., 2020; Zhang G. Q. et al., 2021). Early meta-analyses were based almost entirely on observational studies, and indicated a 29–35% reduced risk of AD in MHT users (Yaffe et al., 1998; LeBlanc et al., 2001). However, the large majority of women in those studies had started MHT before they experienced natural or surgical menopause, generally used estrogen-only therapy (typically CEEs), and stopped using MHT after age 60. As such, the hypothesis that MHT protects against AD was developed based on studies of estrogen-only therapy beginning in early post-menopause (or prior) and stopping a few years post-menopause. In fact, MHT initiated more than 10 years after menopause did not protect against AD (Zandi et al., 2002). Rather, women who initiated MHT between ages 61 and 68 had about double the risk of developing AD as compared to those who had begun MHT at younger ages (Zandi et al., 2002). Today, although results are still mixed, MHT use remains more consistently associated with reduced risk of AD or all-cause dementia as compared to placebo and/or lack of use, especially for estrogen-alone therapy, although all reports indicated substantial heterogeneity and large variability (Hogervorst et al., 2000; Lethaby et al., 2008; O’Brien et al., 2014; Song Y. J. et al., 2020; Zhang G. Q. et al., 2021). As possible biases and lack of control for potential confounders limit interpretation of these studies, more work is warranted to better clarify the role of MHT for AD prevention and preservation of cognitive function.

There is some evidence that MHT may facilitate maintenance of some aspects of cognition when initiated in early post-menopause or prior. Verbal memory is consistently seen to be maintained or sometimes enhanced with estrogen-alone treatment. A review of randomized, placebo-controlled trials of MHT and verbal memory indicate a beneficial effect of estrogen alone therapy in women younger than age 65, especially surgically post-menopausal cases (Maki and Sundermann, 2009). Additionally, different forms of progestogen may have different effects, with negative effects of CEE/MPA on verbal memory in younger women (Maki et al., 2007). There is also indication of positive, yet mild effects of MHT on learning and processing speed (Maki and Sundermann, 2009). Effects vary, however, with MHT type and timing, and there are individual differences, in particular related to time since menopause, type of menopause, and overall neurocognitive health prior to menopause.

Clinical trials using brain scans as endpoints lend support to the hypothesis that both age at treatment initiation and type of MHT are important factors to consider. As summarized in Table 3, the first generation of neuroimaging studies of MHT indicated a generally stimulating or preserving effects of MHT on CBF and CRMglc (Eberling et al., 2000; Maki and Resnick, 2000; Słopień et al., 2003; Rasgon et al., 2005b, 2014; Silverman et al., 2011). Among for women at risk for AD, PET studies provided evidence of differential changes in CMRglc as related to MHT use (Rasgon et al., 2005b, 2014; Silverman et al., 2011). A 2-year longitudinal study showed that non-users exhibited significant CMRglc declines in PCC, whereas MHT users did not exhibit significant CMRglc changes (Rasgon et al., 2005b). Two subsequent prospective, randomized clinical trials investigated post-menopausal women who were taking estrogen-alone MHT for at least 1 year prior to enrollment in the study, and were then randomized to continue or discontinue therapy. Over a 2-year period, women randomized to continue MHT exhibited a relative preservation of frontal and parietal CMRglc as compared with those randomized to discontinue MHT (Silverman et al., 2011; Rasgon et al., 2014). In addition, those continuing unopposed estradiol-based MHT showed additional preservation of CMRglc in PCC and precuneus (Rasgon et al., 2014). Additionally, unopposed MHT use was associated with higher CMRglc in frontal and temporal cortices, as well as better cognitive performance, as compared to opposed MHT, suggesting regionally specific neuroprotective effects (Eberling et al., 2000; Maki and Resnick, 2000; Silverman et al., 2011).

**TABLE 3:** Clinical trials of menopausal hormone therapy (MHT) effects on neuroimaging outcomes.

Study	Participants	Age at baseline, years	MHT type	Imaging modality	Cognitive measures	Study design	Main findings
Shaywitz et al. (1999)	46 POST (last menstrual period > 5 months before enrollment)	33–61	1.25 mg CEE vs. placebo	fMRI	Verbal memory	∼2-month randomized, double-blind, placebo-controlled crossover study	Greater activation in inferior parietal lobule and superior frontal gyrus during verbal and non-verbal retrieval task in treated vs. placebo phase
Joffe et al. (2006)	50 PERI and POST (26 estrogen-treated and 24 placebo-treated women)	40–60	26 tE2 0.05 mg users vs. placebo	fMRI	CVLT, WMS-R, Rey-Osterreith Complex Figure Test	3-month randomized double-blind, placebo-controlled study	Fewer perseverative errors during verbal recall task in MHT users vs. placebo Greater activity in inferior frontal and parietal cortex during verbal memory task in MHT users vs. placebo Greater activity in frontal cortex, posterior cingulate and parietal cortex during spatial memory tasks in MHT users vs. placebo Greater activation in left posterior parietal and left inferior frontal cortices during verbal recall and visual memory task, respectively, in placebo vs. MHT users
Smith et al. (2006)	10 POST [7 (3) years since menopause]	50–60	5 μg ethinyl estradiol and 1 mg norethindrone acetate vs. placebo	fMRI	Visual Delayed Matching to Sample Task	3-month randomized, double-blind, placebo-controlled crossover study	MHT users exhibited higher bilateral prefrontal cortex activation vs. placebo No difference in task performance between active and placebo phase
Coker et al. (2009)	1,403 POST	65–79	257 CEE users, 436 CEE + MPA users, 710 placebo	Structural MRI	Modified Mini-Mental State Exam	Randomized, double-blind, placebo-controlled study (analysis of 1–6 years post-treatment)	No group differences in ischemic lesion volume
Persad et al. (2009)	10 POST	56–60	5 μg ethinyl estradiol + 1 mg norethindrone acetate vs. placebo	fMRI	Verbal memory	3-month randomized, double-blind, placebo-controlled crossover study	Greater activation in left and medial PFC, dorsal anterior cingulate, posterior cingulate, and left parietal cortex in MHT vs. placebo No group differences for verbal memory performance
Resnick et al. (2009)	1,403 POST	65–79	436 0.625 mg CEE with 2.5 mg MPA users vs. 257 0.625 mg CEE alone users vs. 710 placebo	Structural MRI	Modified Mini-Mental State Exam	Randomized, double-blind, placebo-controlled study (analysis of 1–6 years post-treatment)	Reduced hippocampus, frontal cortex, and global GMV in MHT users with large ischemic lesion volume Reductions in hippocampus GMV greatest in MHT users with low baseline cognitive scores
Dumas et al. (2010)	20 POST	59 (6)	10 users 1 mg oral 17β-estradiol vs. placebo	fMRI	Visual verbal n-back task (working memory)	3 month randomized, double-blind, placebo-controlled study	Greater BOLD signal in frontal cortex and precuneus during high word-load condition in MHT users vs. placebo No group differences in performance
Love et al. (2010)	10 POST	56–60	5 μg ethinyl estradiol + 1 mg norethindrone acetate vs. placebo	fMRI	Emotional processing task	3 month randomized, double-blind placebo-controlled crossover study	Greater activation to negative stimuli in left occipital cortex, right precentral gyrus, PCC, and bilateral orbitofrontal cortex in MHT vs. placebo Reduced activation to negative stimuli in DLPFC, postcentral gyrus, and dorsal anterior cingulate in MHT vs. placebo Reduced activation to positive stimuli in left medial frontal cortex in MHT vs. placebo
Davison et al. (2013)	13 POST (no more than 5 years of amenorrhea at randomization)	49–55	6 E_2_D users (continuous-combined estradiol 1 mg/drospirenone 2 mg) vs. placebo	fMRI	Visual attention/vigilance, psychomotor function/speed of processing, paired associates, list learn and recall, Groton Maze learning task and recall	6 month randomized, triple-blind placebo-controlled study	No significant group difference in BOLD signal during verbal fluency or mental rotation tasks No group difference for verbal fluency or mental rotation task performance Higher detection speed in placebo vs. MHT group
Coker et al. (2014)	729 POST	65+	127 CEE, 229 CEE + MPA, 373 placebo	Structural MRI	–	Randomized, double-blind placebo-controlled study (analysis of 1–3 to 6–7 years after treatment)	No group differences in brain or ventricular volume change Smaller frontal GMV in both treated groups vs. placebo at baseline CEE treated patients with a history of cardiovascular disease had greater increases in WMHV and total brain lesion volume No effects of MHT formulation
Kranz et al. (2014)	30 POST	47–64	10 oral estradiol users, 10 oral estradiol + micronized progesterone, 10 placebo	5-HT_1_A PET	–	56–98 day randomized, double-blind, placebo-controlled study	No group differences for 5-HT_1_A serotonin receptor binding
Rasgon et al. (2014)	45 POST (28 continued MHT, 17 discontinued MHT following an average of 10 years of use)	50–65	16 17β-estradiol users (12 with concurrent progestin), 12 CEE users	FDG-PET	–	2 year Randomized, double-blind placebo-controlled study	Greater rates of CMRglc decline in medial PFC, left frontoparietal area, and right inferior parietal cortex in women who discontinued MHT vs. women continuing MHT In ApoE4 non-carriers, greater rates of CMRglc decline in medial PFC and left temporo-occipital area in women who discontinued MHT vs. women continuing MHT Women who discontinued 17β-estradiol, CMRglc decline was greatest in precuneus and PCC, while women who continued 17β-estradiol exhibited no CMRglc decline in precuneus or PCC bilaterally Women who continued CEE exhibited CMRglc decline in bilateral precuneus and PCC Greater rates of CMRglc decline in precuneus and PCC with continuation of 17β-estradiol or CEE with concurrent progestin
Thomas et al. (2014)	13 PERI	48–55	Micronized oral 17β-estradiol subsequently combined with progesterone and placebo	fMRI	Reward processing	4 month randomized, double-blind crossover study	Greater putamen and PFC activity during reward processing in treated group vs. placebo, which correlated with estradiol levels
Berent-Spillson et al. (2015)	29 PERI and POST (6–38 months since last menstrual period)	45–55	1 mg oral estrogen or 200 mg progesterone and placebo	fMRI	Verbal processing and visual working memory	3 month randomized, double-blind crossover study	PFC activity during verbal processing increased by estradiol treatment and decreased by progesterone treatment Decreased HIP activation with estradiol treatment vs. placebo Increased PFC and HIP activation during visual working memory with progesterone treatment
Kantarci et al. (2016a)	68 PERI and POST	52–65	17 oCEE + micronized progesterone, 21 tE2 + micronized progesterone, 30 placebo	PiB PET	CVLT, New York University Paragraphs	4 year randomized, double-blind, placebo-controlled trial	oCEE-treated group had lower CVLT total score compared to placebo Among ApoE4 carriers, tE2 group had lower amyloid burden compared to both placebo and CEE group
Kantarci et al. (2016b)	95 POST (within 5–36 months past their last period)	42–56	29 oCEE + micronized progesterone, 30 tE2 + micronized progesterone, 36 placebo	Structural MRI	Global cognitive function	4 year randomized, double-blind, placebo-controlled trial	Larger ventricular volume in oCEE users vs. placebo Women initiating oCEE later into menopause had larger ventricular volume increases Greater WMHV increase in CEE group at 48 months and in tE2 group at 18 months vs. placebo No group differences in cognition
Zhang et al. (2016)	1,365 POST	65+	254 CEE, 420 CEE + MPA, 691 placebo	Structural MRI	–	Randomized, double-blind, placebo-controlled trial (analysis of 1–3 years post-treatment data)	Reduced frontal GMV in treated groups vs. placebo, especially estrogen-only users No group differences in white matter volume
Albert et al. (2017)	75 POST	51–74	33 high dose oral 17β-estradiol vs. 21 low dose MHT vs. 21 placebo	Structural MRI	–	3 month repeated measures of dose-dependent estradiol treatment vs. placebo	Increased hippocampal GMV with high dose estradiol vs. low dose and vs. placebo
Girard et al. (2017)	12 early POST (6–24 months of amenorrhea after the last menstrual period)	48–55	17β estradiol vs. 17β estradiol + progesterone vs. placebo	fMRI	Cognitive control	4 month randomized, double-blind crossover study	Greater PFC and ACC activation during task switching than in the control condition in active vs placebo phase No differences in task performance
Kantarci et al. (2018)	75 POST	42–56	20 oCEE + micronized progesterone, 22 tE2 + micronized progesterone, 33 placebo	Structural MRI	Global cognitive function	3-year follow-up of Kantarci et al. (2016)	Greater WMHV in oCEE group vs. placebo Slower rates of DLPFC volume decline in tE2 group vs. placebo No group differences in ventricular volumes or cognition
Jayachandran et al. (2020)	95 PERI and POST (within 6 months to 3 years past the last menstrual period)	42–59	29 oCEE + micronized progesterone, 30 tE2 + micronized progesterone, 36 placebo	Structural MRI (WMHV)	–	4 year randomized, double-blind, placebo-controlled trial	No difference between groups for WMHV changes over time
Kling et al. (2020)	78 PERI and POST	42–58	23 oCEE + micronized progesterone, 24 tE2 + micronized progesterone, 31 placebo	Structural MRI	–	4 year randomized, double-blind, placebo-controlled trial	In both treated groups, a greater increase in estrone (E1) associated with smaller increase WMH volume vs. placebo In tE2 group, greater decreases in FSH associated with smaller WMHV increases

*ACC, anterior cingulate cortex; ApoE, apolipoprotein E; BOLD, blood-oxygen-level-dependent; CMRglc, cerebral metabolic rates of glucose; CVLT, California Verbal Learning Test; DLPFC, dorsolateral prefrontal cortex; fMRI, functional MRI; GMV, gray matter volume; HIP, hippocampus; MHT, menopausal hormone therapy; MPA, medroxyprogesterone acetate; MRI, magnetic resonance imaging; oCEE, oral conjugated equine estrogen; PCC, posterior cingulate cortex; PERI, peri-menopausal; PFC, prefrontal cortex; POST, post-menopausal; rsfMRI, resting state fMRI; tE2, transdermal 17β-estradiol; WM, white matter; WMHV, white matter hyperintensity volume; WMS-R, Wechsler Memory Scale-Revised.*

Structural MRI studies reported less consistent evidence of protective effects of MHT. Some report greater GM volumes in MHT users versus non-users (Erickson et al., 2005; Boccardi et al., 2006; Lord et al., 2008) or versus placebo (Eberling et al., 2003; Albert et al., 2017), mostly localized in frontal and temporal cortices, and hippocampus. In some studies, hippocampal volume was positively linked to verbal memory in treated post-menopausal women (Zhang et al., 2016; Braden et al., 2017). However, there are just as many contradictory reports showing decreased frontal GM volume in MHT users versus non-users (Coker et al., 2014; Ryan et al., 2014; Zhang et al., 2016), and decreased hippocampal volume in MHT users versus non-users (Greenberg et al., 2006; Low et al., 2006; Resnick et al., 2009) and in MHT users versus placebo, although there was no further decline from 1–3 to 6–7 years post-treatment (Coker et al., 2014). Notably, reports of positive effects of MHT focused on post-menopausal women in their 60s, whereas negative reports included mostly women of advanced age (71–89 years), sometimes with scanning conducted years after MHT ended. Additionally, two MRI studies showed no differences comparing current or past MHT users to non-users (Ryan et al., 2014; Braden et al., 2017). However, these studies were based on longitudinal WHIMS data collected several years after MHT cessation, and grouped users of estrogen-only and combined therapies, therefore not taking into account possible effects of MHT formulation (Ryan et al., 2014; Braden et al., 2017).

There are also reports of increased white matter hyperintensities with MHT use (Kantarci et al., 2016b) although results on this are mixed (Coker et al., 2014; Zhang et al., 2016) suggesting that effects of MHT on WMH are either small or moderated by confounders, such as age and overall cardiovascular health before treatment.

In addition, randomized controlled trials that incorporated fMRI indicated a higher activation of fronto-cingulate regions and hippocampus during verbal, non-verbal and spatial working memory tasks, although results are not always consistent (Shaywitz et al., 1999; Joffe et al., 2006; Smith et al., 2006; Dumas et al., 2010; Davison et al., 2013; Thomas et al., 2014; Berent-Spillson et al., 2015; Girard et al., 2017). Since these studies reported MHT-related effects in absence of differences in cognitive performance, it remains unclear whether higher activation during task performance reflects a beneficial response or a less efficient use of neuronal resources (Shaywitz et al., 1999; Thomas et al., 2014; Girard et al., 2017).

Overall, brain imaging studies of MHT suggest a putative positive role of estrogen against regional cerebral atrophy and metabolic decline, with an advantage of unopposed over combined MHT (Silverman et al., 2011; Rasgon et al., 2014), and of transdermal estradiol over oral CEE (Resnick et al., 2009; Zhang et al., 2016; Kantarci et al., 2018). However, brain imaging data suffers from several limitations (Comasco et al., 2014). Most studies are statistically under-powered due to relatively small samples and high heterogeneity, including differences in study design (controlled randomization vs. cross-sectional trials, parallel vs. cross-over design, baseline vs. placebo control state), different duration of MHT use, different routes of administration and posology/dose, and different type of therapy (unopposed vs. combined MHT), differences in the timing of initiation with respect to age and/or the menopausal transition, as well as use of different neuroimaging techniques, different neuropsychological paradigms in activation studies, and different processing and analysis pipelines.

In conclusion, active debate remains on whether MHT has value for neuroprotection. Natural history studies and some clinical trials suggest that MHT may support cognition and brain function in peri-menopausal and recently post-menopausal women. However, most studies demonstrating benefits are based on observational studies, or studies of younger women which may have better captured the critical window for MHT action vs. larger clinical trials of older post-menopausal women. Observational studies are subject to bias as women who choose to use MHT have in general higher education, and tend to have healthier lifestyles and better overall health before and after taking MHT than women who do not (Matthews et al., 1996). Taking MHT may therefore be associated with a healthier lifestyle which in turn might be driving cognitive function. In addition, despite estrogen’s biologically plausible mechanisms for supporting brain aging, most reviews have concluded that many observational studies and clinical trials are limited by methodological problems such as small size and short duration, and display substantial heterogeneity.

## Conclusion

Understanding sex-driven effects of ovarian hormones on dementia risk is a crucial step toward development of precision medicine strategies for AD prevention. In recent years, significant progress has been made in discovering how ovarian steroid hormones influence cognitive aging, prompted in part by advancements in the research on sex differences in AD. Across the female lifespan, there is compelling evidence that estradiol levels influence brain structure, function, and biochemistry in many regions affected by AD. There are also increasing indications of complex interactions of estradiol with other sex hormones, chiefly progesterone and androgens.

In this review we examined the effects of puberty, the menstrual cycle, hormonal contraceptives, menopause, and MHT on cognitive aging and neuroimaging biomarkers of AD. While this field is still in its infancy, there is increasing evidence for associations between indicators of estrogen exposure, such as pubertal timing, menstrual cycle frequency, number of pregnancies, and OC use, and cognitive function over the course of a woman’s life (Egan and Gleason, 2012; Li et al., 2016).

More work has been done to investigate changes in cognition and AD biomarkers during the transition to menopause, and more so as due to MHT use. Clinical studies indicate a dip in cognitive performance, mostly verbal memory, during peri-menopause, possibly followed by a rebound post-menopause. Significant heterogeneity has been noted as related to age, menopause status, use of MHT, and genetic risk factors. Hardly any clinical studies analyzed data in relation to women’s existing genetic predisposition to AD or other neurological conditions. On the other hand, neuroimaging studies of midlife women at genetic risk for AD have provided robust evidence for emergence of AD endophenotypes with onset in peri-menopause among natural cyclers (Mosconi et al., 2017, 2018a,b, 2021; Rahman et al., 2020). Surgically induced menopause is also associated with a higher risk of AD, especially in presence of an earlier age at oophorectomy (Bove et al., 2014). Across studies, the risk of AD is over 30% higher following hysterectomy alone, and over two times higher in presence of oophorectomy relative to spontaneous menopause. For contrast, women’s risk of AD is increased 4- and 12–15-fold with one or two ApoE4 alleles, respectively (Riedel et al., 2016). More work is needed to examine the combined effects of ApoE4 and hysterectomy/oophorectomy status prior to menopause on AD biomarkers, and whether the associations are modified by MHT use.

Menopause hormone therapy use has been heavily scrutinized due to the disparity between basic science, observational studies, and large randomized clinical trials. Overall, MHT action on brain is dependent on multiple factors, including chronological age, stage of reproductive aging, duration of hypogonadism, and presence of symptoms, as well as the formulation of MHT, route of administration, and the health status of the brain. Currently, MHT is not indicated to alleviate cognitive complaints or for AD prevention. However, some argue that MHT given to healthy peri-menopausal and early post-menopausal women under age 60 for about 5 years may be recommended for support of cognitive function with careful consideration of other risks (Stuenkel et al., 2015; Baber et al., 2016). There is mounting evidence that MHT use during early menopause, and in presence of symptoms, may help sustain neurological health and reduce the risk of AD (Brinton, 2008), whereas MHT initiated >5 years after menopause may be less beneficial if not detrimental as in the case of combined therapy (Shumaker et al., 2003). Personalized physician advice which takes into consideration key factors including age, menopausal stage, symptoms, and comorbidities, may offer a greater look at how MHT impacts AD risk as compared to the one-size-fits-all approach of randomized clinical trials, and argues for a precision medicine approach to MHT use (Kim and Brinton, 2021; Kim et al., 2021). More research is warranted to further understand this critical window of estrogen sensitivity.

Previous work has shown that, *in vitro* and *in vivo*, ApoE expression can be differentially regulated either by 17-beta-estradiol or specific agonists, depending on activation of ER subtypes (Wang et al., 2006). These data suggest that use of ER-selective ligands might provide therapeutic benefit to reduce AD risk by decreasing ApoE expression in ApoE4 allele carriers. Moreover, because ERβ promotes estrogen-mediated neuronal plasticity and memory function, a formula that selectively targets ERβ may be a novel and plausible solution for menopause-related vasomotor symptoms and cognitive impairment. In 2022, we obtained NIH funds to carry out a Phase IIb randomized, placebo-controlled clinical trial testing the efficacy of PhytoSERM, a selective estrogen receptor beta (ERβ) modulator comprised of three phytoestrogens: genistein, daidzein, and S-equol (Zhao et al., 2009), for AD prevention in midlife women. The PhytoSERM formulation has been shown to promote estrogenic action in brain while remaining largely inactive or inhibitory in reproductive tissue (Zhao et al., 2009). The initial phase Ib/IIa clinical trial (ClinicalTrial.gov ID: NCT01723917) demonstrated safety and established the pharmacokinetics profile of PhytoSERM (Wang et al., 2020a). Results of the ongoing Phase IIb trial will become available by 2026.

In conclusion, ovarian steroid hormones are long overlooked but critical contributors to brain aging and AD risk. While the neurobiological consequences of hormonal activity have only begun to be understood, converging evidence supports a role for cumulative estrogen exposure in reducing risk of developing AD later in life. This strongly argues for continued examination of sex hormones and reproductive history factors in AD prevention strategies for women. There is an urgent need for prospective epidemiological, clinical and biomarkers studies with data taken at several time-points starting at midlife that examine the associations between lifetime estrogen exposure and neurological function in later life. Understanding the dynamic interplay between sex, chronological aging, endocrine aging, and additional AD risk factors is crucial to inform and justify primary precision-medicine strategies for AD prevention.

## Author contributions

LM and SJ discussed the concepts and wrote the manuscript. ES, GJ, CB, JD, SP, and RD reviewed the literature and provided critical revision of the manuscript for important intellectual content. All authors contributed to the article and approved the submitted version.

## Conflict of interest

The authors declare that the research was conducted in the absence of any commercial or financial relationships that could be construed as a potential conflict of interest.

## Publisher’s note

All claims expressed in this article are solely those of the authors and do not necessarily represent those of their affiliated organizations, or those of the publisher, the editors and the reviewers. Any product that may be evaluated in this article, or claim that may be made by its manufacturer, is not guaranteed or endorsed by the publisher.
